# Identification of robust genetic signatures associated with lipopolysaccharide-induced acute lung injury onset and astaxanthin therapeutic effects by integrative analysis of RNA sequencing data and GEO datasets

**DOI:** 10.18632/aging.104042

**Published:** 2020-09-23

**Authors:** Kaimin Mao, Wei Geng, Yuhan Liao, Ping Luo, Hua Zhong, Pei Ma, Juanjuan Xu, Shuai Zhang, Qi Tan, Yang Jin

**Affiliations:** 1 Department of Respiratory and Critical Care Medicine, NHC Key Laboratory of Pulmonary Diseases, Union Hospital, Tongji Medical College, Huazhong University of Science and Technology, Wuhan 430022, Hubei, China; 2 Center for Translational Medicine, Union Hospital, Tongji Medical College, Huazhong University of Science and Technology, Wuhan 430022, Hubei, China; 3 College of Life Sciences, Wuhan University, Hubei Province, Wuhan, 430072, China

**Keywords:** acute lung injury, genetic signatures, RNA sequencing, integrative analysis, astaxanthin

## Abstract

Acute lung injury (ALI) and acute respiratory distress syndrome (ARDS) are life-threatening clinical conditions predominantly arising from uncontrolled inflammatory reactions. It has been found that the administration of astaxanthin (AST) can exert protective effects against lipopolysaccharide (LPS)-induced ALI; however, the robust genetic signatures underlying LPS induction and AST treatment remain obscure. Here we performed a statistical meta-analysis of five publicly available gene expression datasets from LPS-induced ALI mouse models, conducted RNA-sequencing (RNA-seq) to screen differentially expressed genes (DEGs) in response to LPS administration and AST treatment, and integrative analysis to determine robust genetic signatures associated with LPS-induced ALI onset and AST administration. Both the meta-analyses and our experimental data identified a total of 198 DEGs in response to LPS administration, and 11 core DEGs (*Timp1, Ly6i, Cxcl13, Irf7, Cxcl5, Ccl7, Isg15, Saa3, Saa1, Tgtp1,* and *Gbp11*) were identified to be associated with AST therapeutic effects. Further, the 11 core DEGs were verified by quantitative real-time PCR (qRT-PCR) and immunohistochemistry (IHC), and functional enrichment analysis revealed that these genes are primarily associated with neutrophils and chemokines. Collectively, these findings unearthed the robust genetic signatures underlying LPS administration and the molecular targets of AST for ameliorating ALI/ARDS which provide directions for further research.

## INTRODUCTION

Acute respiratory distress syndrome (ARDS) is an acute inflammatory lung injury, associated with increased pulmonary vascular permeability, increased lung weight, and loss of aerated lung tissue [1]. Its less severe form is acute lung injury (ALI). Most patients need mechanical ventilation for support. The initial acute or exudative phase of ALI/ARDS is characterized by the rapid onset of dyspnea, hypoxemia, respiratory failure, and bilateral infiltrates on chest radiographs that are consistent with pulmonary edema [2]. ALI/ARDS is common and has been associated with several clinical disorders, such as sepsis; pneumonia; aspiration of gastric contents, saltwater, or freshwater; major trauma; transfusion of blood products; acute pancreatitis; and drug reactions (for example, reactions to lipopolysaccharide) [3]. In the past 50 years, considerable progress has been made in understanding the epidemiology, pathogenesis, and pathophysiology of ARDS. However, ARDS is being increasingly recognized as a heterogeneous syndrome, generating momentum for the identification of clinical and biological features for classifying patients into subphenotypes that might be more responsive to specific therapies.

Lipid A (endotoxin), the hydrophobic anchor of lipopolysaccharide (LPS), is a glucosamine-based phospholipid that makes up the outer monolayer of the outer membranes of most Gram-negative bacteria [4]. In recent years, LPS, which has been most widely used in drug-associated ALI models, can effectively induce a neutrophilic inflammatory response accompanied by an increase in intrapulmonary cytokines. Many studies have shown that oxidative stress plays a major role in the pathogenesis of lung injury in a murine model of ALI induced by lipopolysaccharide (LPS) [5–7]. In response to the increased formation of reactive oxygen species (ROS), thioredoxin interacting protein (TXNIP) detaches from thioredoxin (Trx), binds to the nucleotide-binding domain-like receptor protein 3 (NLRP3), and then activates NLRP3 inflammasome [8]. The activation of the NLRP3 inflammasome results in the maturation and release of pro-inflammatory cytokines, such as interleukin-1β (IL-1β), which further aggravates the production of inflammatory cytokines (tumor necrosis factor-α (TNF-α), IL-6, inducible nitric oxide synthase (iNOS), and cyclooxygenase-2 (COX2)) and induces oxidative stress [9–11].

Astaxanthin (AST) is a lipid-soluble, red-orange-colored xanthophyll carotenoid synthesized by many microorganisms and various types of marine life. The main producers of natural AST are microalgae and fungi. Aquatic animals such as salmon, red seabream, shrimp, lobster and crayfish, which feed on AST-producing organisms, are significant dietary sources of AST for humans [12–14]. It has been revealed that AST can prevent inflammatory processes by blocking the expression of pro-inflammatory genes as a consequence of suppressing nuclear factor kappaB (NF-κB) activation [15]. Some studies also suggested that AST has a dose-dependent ocular anti-inflammatory effect, through the suppression of NO, PGE2, and TNF-alpha production, which is achieved by directly blocking NOS enzyme activity [16]. Furthermore, AST has great therapeutic value for lung disease, such as an antifibrotic effect against the promotion of myofibroblast apoptosis based on dynamin-related protein-1 (Drp1)-mediated mitochondrial fission in vivo and in vitro [17] and anti-inflammatory effect against LPS-induced ALI, as mentioned above [18, 19]. However, at the transcriptional level, the mechanism of action of AST in the treatment of ALI-/ ARDS- remains unclear. Therefore, we hope to explore the molecular targets of AST against ALI- / ARDS- through further research, with the purpose of providing a new alternative for the clinical treatment of this acute lung disease.

## RESULTS

### Data processing and meta-analysis of the five microarrays

**Figure 1:**
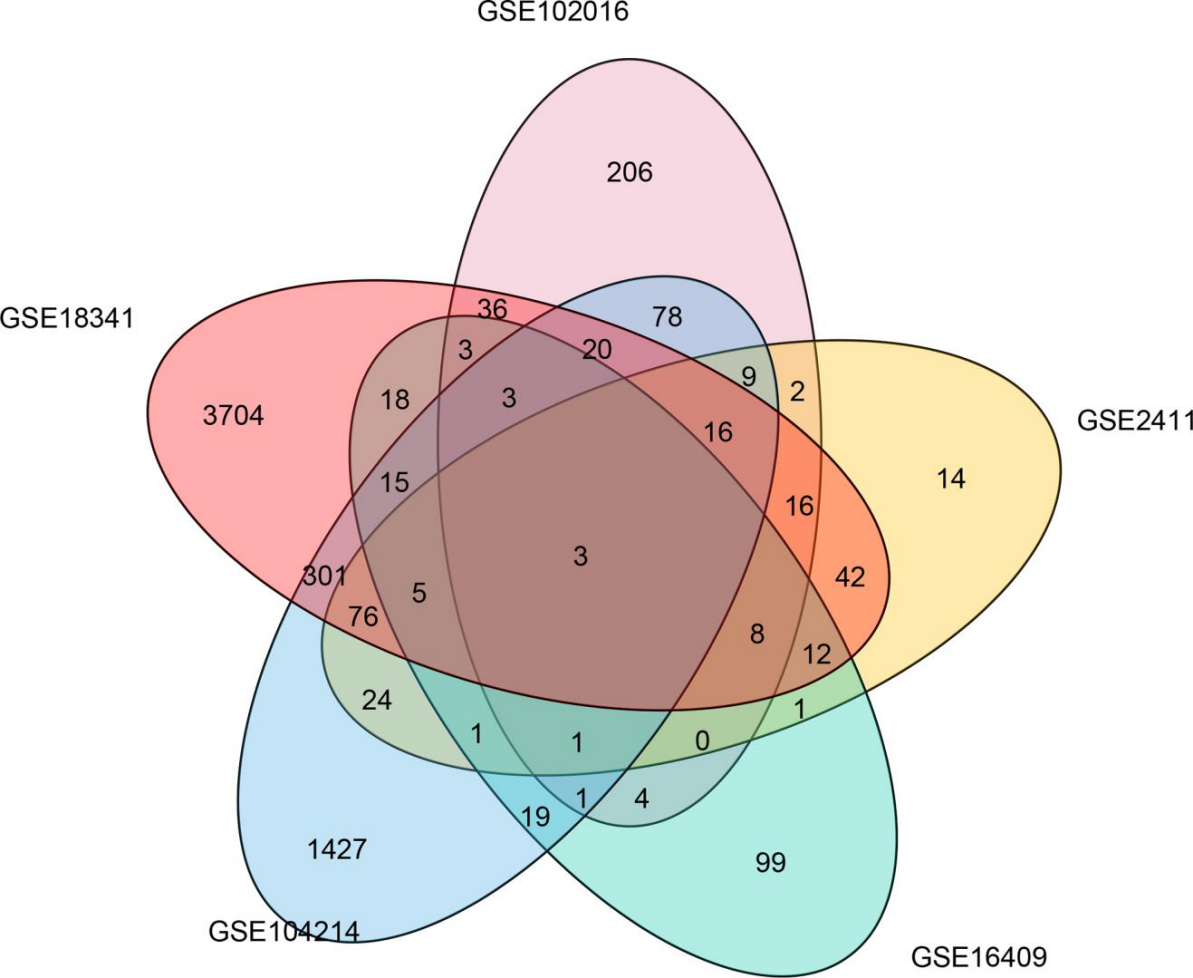
**Common differentially expressed genes associated with LPS induction in five microarray studies.** A Venn diagram was constructed to show the overlapping differentially expressed genes associated with LPS induction identified in five microarray studies.

To determine the common molecular signatures underlying LPS-induced mouse ALI initiation, five microarray datasets were obtained from corresponding independent studies. The characteristics of the studies composing the five gene expression compendiums are listed in Table 1 and Supplementary Table 1. We extracted and annotated the five microarrays, which yielded a collection of 4093 unique genes from 64 samples, including 26 control and 36 LPS-induced ALI mice. Before the meta-analysis study, we comprehensively analyzed the five datasets by identifying the differentially expressed genes in each data set and evaluated overlapping significant genes. The overlapping results were used to generate a Venn diagram (Figure 1), and three genes (*Ccl12, Zbp1,* and *Cxcl13*) were identified in the common region, suggesting that these three genes were significantly correlated with LPS management in mice in the five datasets.

**Table 1:** Characteristics of studies composing the five gene expression compendium.

**Study**	**Dataset**	**Platform**	**Region**	**Types of sample**	**Sample (Control)**	**Sample (LPS-ALI)**
[60]	GSE102016	Affymetrix Mouse Gene 1.1 ST Array	Maastricht, Limburg Netherlands	Mouse lung tissue	3	3
[58]	GSE2411	Affymetrix Mouse Expression 430A Array	Seattle, WA USA	Mouse lung tissue	6	6
[62]	GSE16409	Amersham CodeLink UniSet Mouse 20K I Bioarray	Durham, NC USA	Mouse lung tissue	3	9
[59]	GSE104214	Agilent-028005 SurePrint G3 Mouse GE 8x60K Microarray	New Haven, CT USA	Mouse lung tissue	6	11
[61]	GSE18341	Affymetrix Mouse Genome 430 2.0 Array	Seattle, WA USA	Mouse lung tissue	8	8

**Figure 2:**
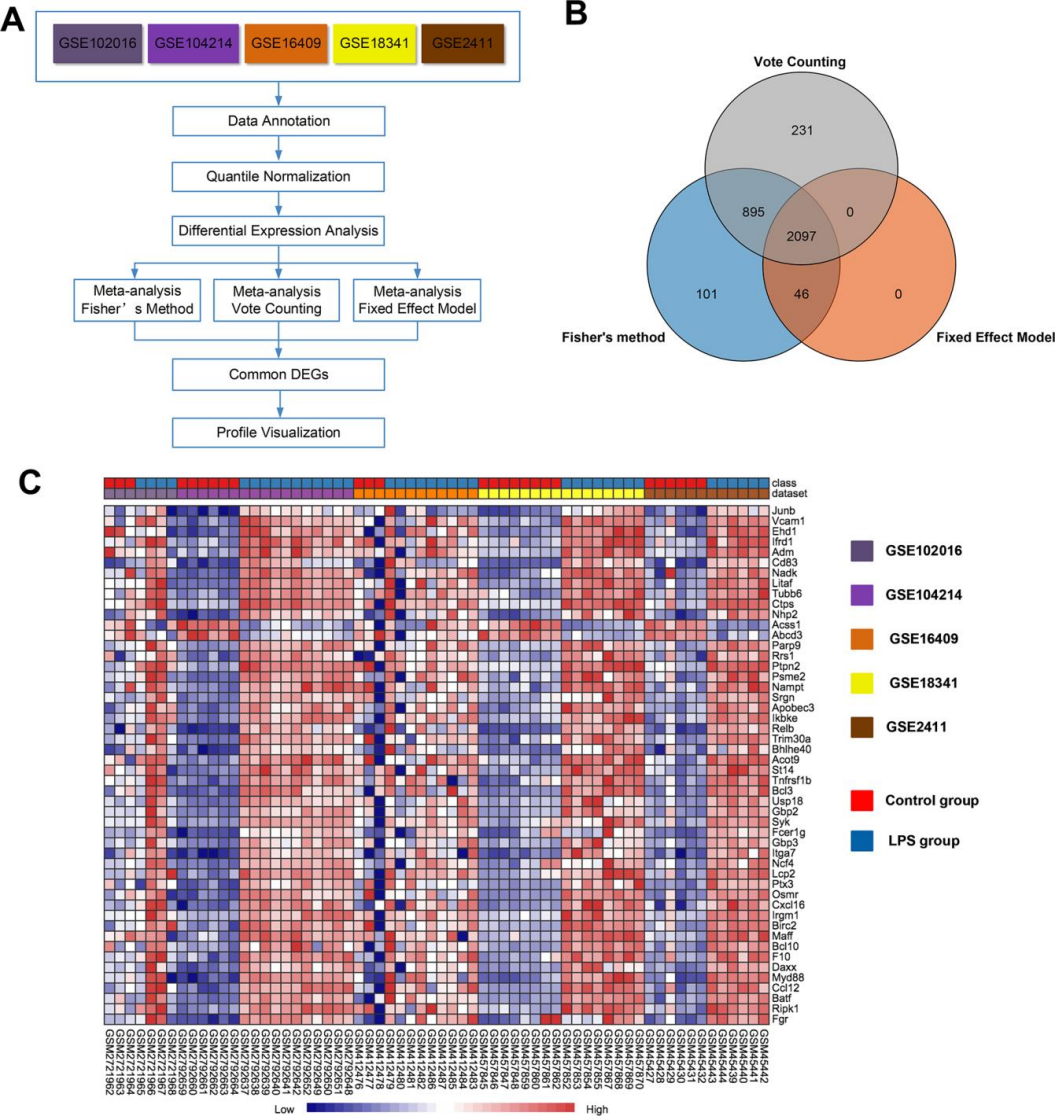
**Meta-analysis of five microarrays to identify genes related to LPS induction.** (**A**) Flowchart of the meta-analysis approach. (**B**) Venn diagram of differentially expressed genes identified by the meta-analysis using Fisher's method, the vote-counting method, and a fixed-effect model. (**C**) Heat map visualization of the top 50 consistently differentially expressed genes (either completely upregulated or downregulated) across the five microarrays identified by the meta-analysis (row-wise comparison).

Then, we performed a meta-analysis using NetworkAnalyst (http://www.networkanalyst.ca), which is a comprehensive web-based tool designed to perform meta-analyses of gene expression data [20]. An overview outlining the procedure of the analysis is presented in Figure 2A. Using three meta-analysis approaches, namely Fisher's method, Fixed effect model and Voting count, 3139, 2143 and 3223 differentially expressed genes, respectively, were identified. Among these genes, 2097 were identified by all three methods (Figure 2B), with 1043 (49.7%) genes being upregulated and 1054 (50.3%) being downregulated in the LPS group compared with the control group. A full list of the 2097 common genes identified by the three meta-analysis methods is presented in Supplementary Table 2. A heat map of the top 50 common DEGs across the five datasets is displayed in Figure 2C. Of note, the 10 top upregulated genes (P<0.05) were *Junb, Vcam1, Ehd1, Ifrd, Adm, Cd83, Nadk, Litaf, Tubb6,* and *Ctps*. The 2 most significantly downregulated genes (P<0.05) among the top 50 common DEGs were *Acss1* and *Abcd3*. The merged data from this meta-analysis are listed in Supplementary Data 1.

### Identification of DEGs associated with AST treatments

**Figure 3:**
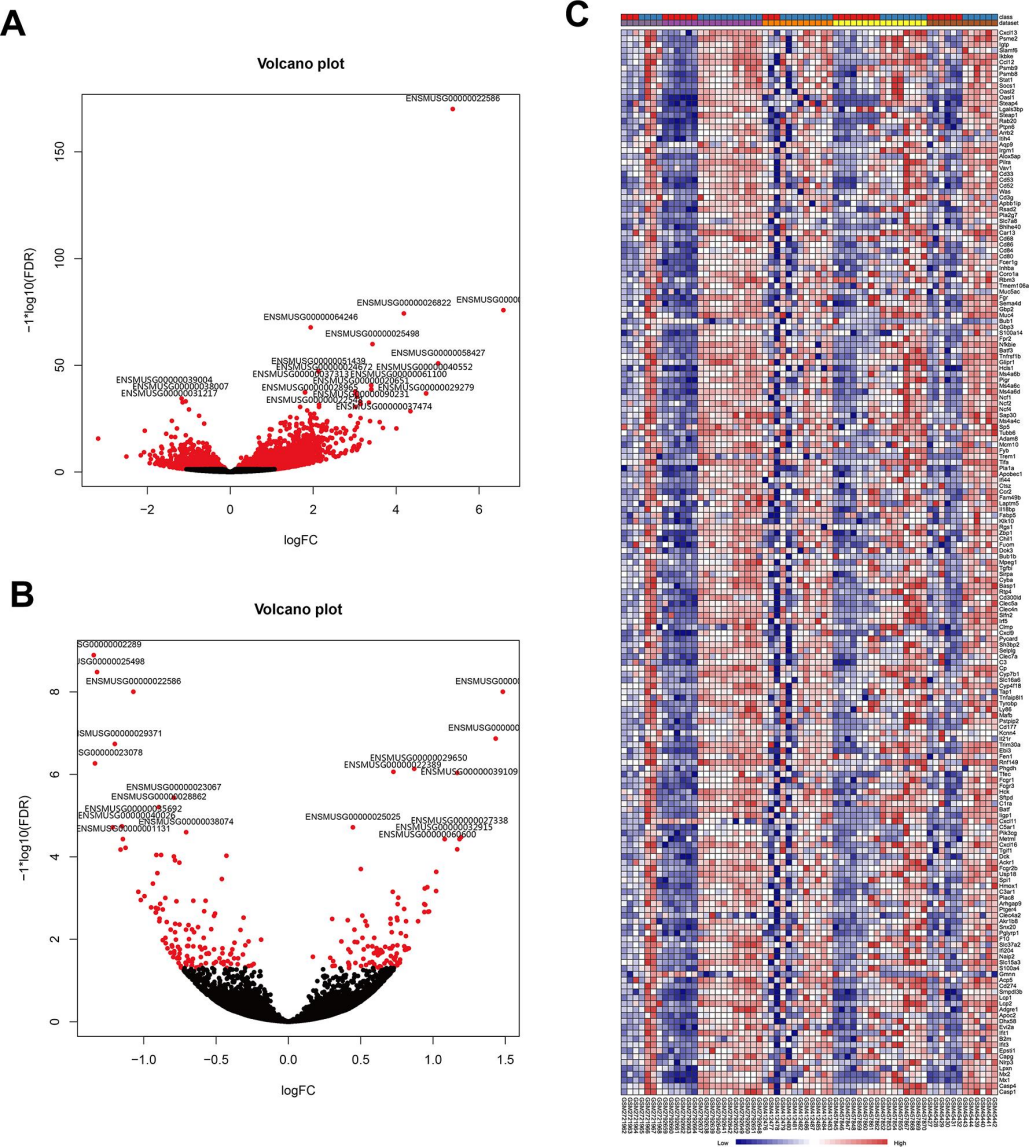
**Visualization of DEGs identified by RNA sequencing and the overlap of the results with the meta-analysis of the five microarrays.** (**A**) Volcano plot representation of the1187 DEGs associated with LPS-induced ALI onset identify by RNA sequencing (control group vs. LPS group). (**B**) Volcano plot representation of the 21 DEGs associated with the AST therapeutic effect of AST identified by RNA sequencing (LPS group vs. AST group). (**C**) Heat map of the 181 overlapping upregulated genes identified by both meta-analysis of published microarrays and our RNA sequencing experiments (control group vs. LPS group).

To further identify the robust expression signatures in LPS-induced ALI and investigate the transcriptional changes resulting from treatment of ALI with AST, we divided mice into three groups, including the control group, LPS group, and AST group. RNA-sequencing (RNA-seq) was performed to profile differentially expressed genes (DEGs) associated with LPS-induced ALI initiation and AST treatment. A total of 1187 DEGs were identified in the LPS-induced ALI group compared with the control group. Among these genes, 989 were significantly upregulated, and 198 were significantly downregulated (Figure 3A and Supplementary Figure 2-1, Supplementary Figure 2-2). A complete list of the differentially expressed genes is provided in Supplementary Table 3. Then, we compared these genes with the DEGs obtained from the above meta-analysis, and generated two heat- maps of the common DEGs across the meta-analysis results and our experimental data, which are displayed in Figure 3C and Supplementary Figure 1. In total, 198 DEGs were detected in both published data and our experimental data, including 181 upregulated and 17 downregulated DEGs.

To explore the therapeutic effect of AST against ALI at the genetic level, we also compared the gene expression profile of the LPS-induced ALI group with that of the AST treatment group. In total, 21 DEGs were identified after AST treatment (Figure 3B and Supplementary Figure 3-1, and Supplementary Figure 3-2), of which 9 were significantly upregulated and 12 were significantly downregulated (Supplementary Table 4). We subsequently integrated the RNA-seq and microarray meta-analysis data, and 11 core DEGs (*Timp1, Ly6i, Cxcl13, Irf7, Cxcl5,* *Ccl7, Isg15, Saa3, Saa1, Tgtp1*, and *Gbp11*) that were upregulated in ALI models and downregulated significantly after AST treatment were identified (Table 2).

**Table 2:** List of eleven core DEGs.

**Gene**	**ENSEMBL ID**	**AST vs LPS**			**LPS vs Control**		
		**log2 Fold Change**	**P-value**	**P-adj**	**log2 Fold Change**	**P-value**	**P-adj**
Timp1	ENSMUSG00000001131	-1.144380002	4.22E-08	3.74E-05	3.079304599	3.48E-26	1.23E-23
Ly6i	ENSMUSG00000022586	-1.07173215	2.35E-12	9.97E-09	5.366650913	4.43E-175	7.5E-171
Cxcl13	ENSMUSG00000023078	-1.337143037	2.23E-10	5.38E-07	1.311102736	0.000340307	0.002635586
Irf7	ENSMUSG00000025498	-1.322720054	3.90E-13	3.31E-09	3.428565104	3.96E-64	1.34E-60
Cxcl5	ENSMUSG00000029371	-1.199834605	6.50E-11	1.84E-07	3.370918298	3.3E-27	1.42E-24
Ccl7	ENSMUSG00000035373	-1.020396206	2.84E-06	0.001119	3.168049161	7.33E-18	8.62E-16
Isg15	ENSMUSG00000035692	-1.151876996	1.41E-08	1.83E-05	2.192186131	5.14E-22	9.46E-20
Saa3	ENSMUSG00000040026	-1.21478413	1.70E-08	1.92E-05	6.587536481	1.63E-80	1.38E-76
Saa1	ENSMUSG00000074115	-1.037241452	1.61E-06	0.000706582	4.344103762	5.13E-32	3.34E-29
Tgtp1	ENSMUSG00000078922	-1.126181149	7.50E-08	6.05E-05	1.653443678	9.49E-09	0.000000246
Gbp11	ENSMUSG00000092021	-1.160260065	9.11975E-08	6.71531E-05	2.140380325	1.16E-09	3.56E-08

### Functional annotation of core DEGs

**Figure 4:**
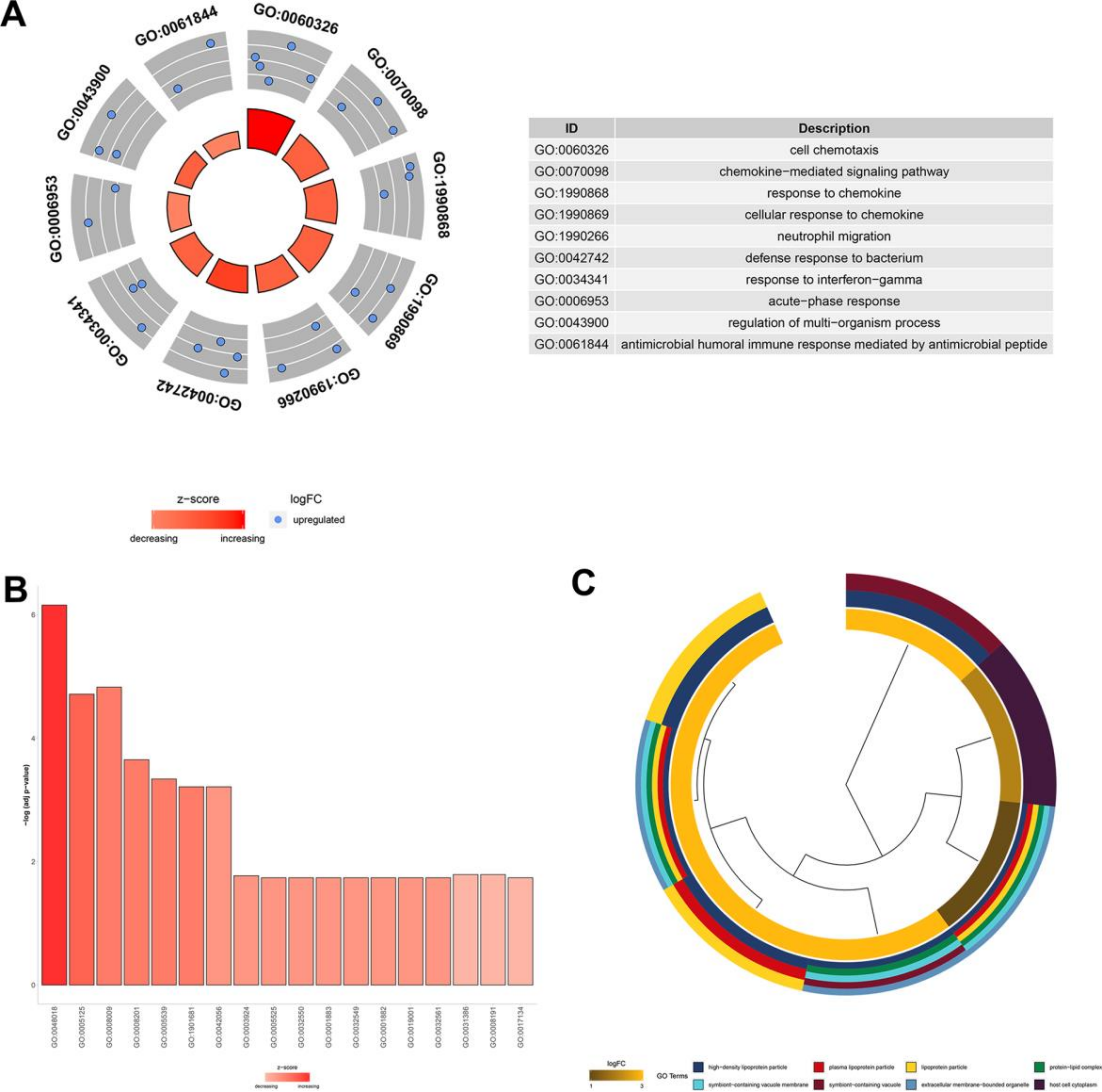
**GO functional analysis of the 11 core DEGs related to the therapeutic effect of AST.** (**A**) Top 10 significantly (P<0.05) enriched terms in biological processes. (**B**) Significantly (P<0.05) enriched terms in molecular functions. (**C**) Top 8 significantly (P<0.05) enriched terms in cellular components.

To understand the function of the 11 core DEGs, GO enrichment analysis including molecular function (MF), biological process (BP) and cellular component (CC) categories (Supplementary Table 5) was performed using the ‘clusterprofile’ package in R [21]. In BP terms, the upregulated genes were associated with “cell chemotaxis,” the “chemokine−mediated signaling pathway,” and “neutrophil migration” (Figure 4A).

Several studies have shown that neutrophil migration and related chemokine network regulation in the lung play roles in the pathogenesis and development of ALI/ARDS [22–24]. In the MF category, the core DEGs were associated with "glycosaminoglycan binding," "chemokine activity," and "receptor-ligand activity" (Figure 4B). Since glycosaminoglycan–cytokine interactions have been reported to support cellular mechanisms that cause acute inflammation [25], AST may affect these interactions by downregulating the genes involved to exert an anti-inflammatory effect. Moreover, DEGs were enriched in the CC category involved in "high−density lipoprotein particles," "symbiont−containing vacuole membranes," and "plasma lipoprotein particles" (Figure 4C).

### Validation of core DEGs

**Figure 5:**
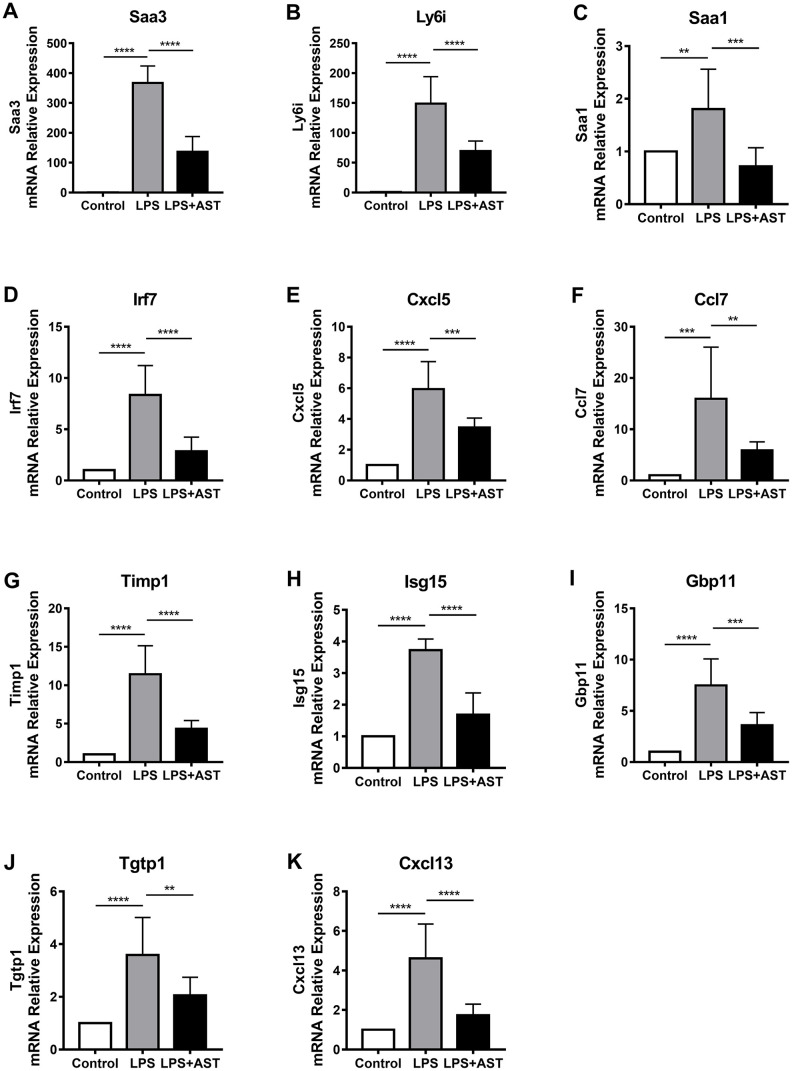
**Changes in the expression of 11 core genes (A-K: *Saa3, Ly6i, Saa1, Irf7, Cxcl5, Ccl7, Timp1, Isg15, Gbp11, Tgtp1,* and *Cxcl13*) in the lungs of an established mouse model.** Quantitative real-time polymerase chain reaction (RT-qPCR) was conducted to quantify the relative expression of the 11 core genes in the control group (n = 4), LPS-induced ALI group (n = 4), and AST treatment group (n = 4). GAPDH was used as an internal control gene. The experiments were performed at least in triplicate. The error bars represent the SEM. Statistical analysis of significant differences between groups was achieved with one-way ANOVA using Prism 7 software. ****p < 0.0001, ***p < 0.001, **p < 0.01, and *p < 0.05 were considered statistically significant.

To further confirm the differences in the expression of the 11 core DEGs (*Timp1, Ly6i, Cxcl13, Irf7, Cxcl5, Ccl7, Isg15,* *Saa3, Saa1, Tgtp1*, and *Gbp11*) among the control group, LPS group, and AST group, we divided mice into three groups and conducted qRT-PCR and IHC verification (Figure 5A–5K, Supplementary Figure 4). The results demonstrate that the relative expression levels of all 11 genes were significantly upregulated in the LPS group compared to the control group. More importantly, the expression levels of the above 11 DEGs, as analyzed by qRT-PCR, were significantly inhibited after the application of AST. Of the 11 core genes, 8 were tested by IHC, and the results were consistent with the qRT-PCR results, which further verifying the data (Supplementary Figure 4). Overall, the RT-qPCR and IHC results were consistent with our integrative RNA-seq analysis and meta-analysis, suggesting the critical role that the 11 core DGEs might play in the mechanism by which AST alleviates ALI/ARDS.

## DISCUSSION

As a life-threatening condition, ALI/ARDS is an underrecognized condition, and its treatment is an unmet medical need. It is thought that inflammatory storm is the key factor in the occurrence of ALI [26], and anti-inflammatory and antioxidant therapy should be the primary objective in ALI/ARDS [27]. To find the conserved genes responsible for LPS-induced ALI initiation and the effects of AST treatment, we identified robust changes in gene expression related to ALI by meta-analysis and RNA-seq using the Gene Expression Omnibus (GEO) database and mice, respectively. Moreover, we performed functional enrichment analysis of core genes using the Gene Ontology (GO) database to explore the possible molecular mechanisms that mediate the therapeutic effect of AST. Before the meta-analysis of the five microarray datasets, we compared the differentially expressed genes in each dataset, and 3 common differentially expressed genes (DEGs) were found in all five datasets: *Cxcl13, Zbp1, and Ccl12*. CXCL13 is abnormally expressed in the lung tissues of patients with idiopathic pulmonary fibrosis (IPF), and its circulating concentration is also highly correlated with the clinical manifestations and disease progression of individual patients. In the lung tissues of patients with IPF, CXCL13 may promote focal infiltration of nonproliferating B cells through the CXCL13-CXCR5 axis [28]. ZBP1 is a host protein that was shown to be an innate sensor of viral infection, regulating cell death, inflammasome activation, and proinflammatory responses in a variety of situations, including infection and embryonic development [29]. A previous study indicated that ZBP1 is abnormally expressed in H1N1-induced pneumonia associated with acute respiratory distress syndrome in mice [30]. CCL12 (MCP1), which is elevated in pulmonary fibrosis, has been reported to mediate fibroblast survival through IL-6 [31]. Since fibroproliferation is initiated early in lung injury, it has been observed that CCL12 is highly expressed in ARDS induced by severe sepsis [32]. To reduce the study bias and increase the statistical power of individual microarray data, we performed a meta-analysis of five microarray gene expression profiles to assess the differentially expressed genes between LPS-induced and control groups. Consequently, 2097 differentially expressed genes (DEGs) were identified using three meta-analysis approaches. To further identify the robust expression signature related to LPS-induced ALI and investigate the transcriptional changes in response to the treatment of ALI by AST, we performed RNA-seq on three groups of mice and integrated the data with the results of the above mentioned meta-analysis. Ultimately, we identified 11 core DEGs that were significantly associated with AST treatment. *Saa3, Ly6i, Saa1, Irf7, Cxcl5, Ccl7, Timp1, Isg15, Gbp11, Tgtp1,* and *Cxcl13* were found to be overexpressed in the LPS group compared with the control group but relatively downregulated in the AST group. Our qRT-PCR and IHC verification of the 11 core genes in the mice suggested that these genes might be the key mediators of the therapeutic effect of AST in ALI/ARDS.

Among the 11 core genes that were differentially expressed in response to AST mediation, two genes are members of the serum amyloid A (SAA) family. SAA is a critical acute-phase protein that is often increased by infection, trauma, cancer, or other causes of inflammation and plays an important role in the regulation of inflammatory responses [33]. Recent studies have indicated that an increased level of SAA is positively correlated with the disease progression of COVID19, and can thus be a sensitive indicator for assessing the severity and prognosis of COVID-19 [34]. In our study, *Saa3* was the most significantly inhibited gene by AST application in LPS-induced ALI mice, and its downregulation was further confirmed by qRT-PCR and IHC. SAA3, the one of three isoforms of SAA expressed in mice, is stimulated intensely in LPS-induced acute systemic inflammation, which is consistent with our findings [35]. High expression of SAA3 in response to acute inflammation may be repressed by an interaction with noncoding RNAs. It has been confirmed that miR-30b-3p may target *Saa3* to protect against LPS-induced ALI [36]. Additionally, lncRNA MALAT1 can also target *Saa3* directly or indirectly to cause many diseases such as inflammation, diabetes and septic cardiomyocytes [37, 38]. SAA1, another member of the SAA gene family, is believed to have a pro-inflammatory effect, and its expression may aggravate tissue inflammation and damage [39]. Removing the N- and C-terminal sequences of SAA1 can switch the protein to an anti-inflammatory role [40]. However, other research has suggested that mice induced to express genetically modified human SAA1 have a partial protective effect against the inflammatory response and lung injury caused by LPS [41]. Moreover, *Saa1* is the direct target of miR-660, which can protect nucleus pulposus cells from TNFa-induced apoptosis in intervertebral disc degeneration [42]. Considering that SAA might act as a biomarker of inflammatory disease, it is possible, that its downregulation induced by AST may partly indicate the anti-inflammatory effect of AST. The deeper molecular mechanism underlying SAA action in response to AST application deserves further exploration.

Interferon regulatory factor 7 (IRF7) is considered the master regulator of IFN-α against pathogenic infections [43]. The excessive activation of IRF7 promotes the development of acute lung injury (ALI) caused by influenza A virus (IAV), and attenuating IRF7 activity can significantly prevent the progression of IAV-induced ALI in model mice [44]. Thus, the present finding that IRF7 was upregulated by LPS and downregulated in response to AST treatment may suggest that of AST protects against ALI.

Regarding how IRF7 regulates IFN production, miRNA may act as an important mediator. Previous research has shown that miR-302c can downregulate IRF3 and IRF7 expression to mediate influenza A virus-induced IFNβ expression [45]. Additionally, miR-144 was shown to reduces the antiviral response by attenuating the TRAF6-IRF7 pathway to alter the cellular antiviral transcriptional landscape [46]. However, whether miRNA-IRF7 interactions are involved in the pharmacological mechanism of AST remains to be further investigated.

Tissue inhibitor of metalloproteinase-1 (TIMP1), a member of TIMP family, is primarily recognized to regulate the degradation of the extracellular matrix by inhibiting the activity of matrix metalloproteinases (MMPs) [47]. It has been reported that an imbalance between MMP9 and TIMP1 plays a pivotal role in the pathogenesis of ARDS mainly through participating in airway remodeling, thus indicating the function of the MMP9/TIMP1 ratio in the evolution of pulmonary fibrosis in ARDS [48]. Indeed, increased systemic levels of TIMP1 were proven to be associated with increased 90-day mortality in ARDS patients according to a large, prospective, multicenter study [49]. Additionally, other research has demonstrated that increased TIMP1 expression promotes an immune response, has a pro-inflammatory effect in the lungs after influenza infection and facilitates an injurious phenotype [50]. The above observations not only support our present results regarding TIMP1 but also provide a considerable explanation for the increase in TIMP1 expression after LPS application. Intriguingly, given that TIMPs are highly expressed in liver fibrosis and that the imbalance of MMPs/TIMPs promotes the progression of fibrosis, Shen et al. found that astaxanthin is able to repress the activation of hepatic stellate cells (HSCs) to ameliorate liver fibrosis through downregulating the expression of NF-κB and TGF-β1 and preserving the balance between MMP2 and TIMP1 [48]. Hence, it is reasonable to further investigate whether there is a similar mechanism by which AST downregulates the expression of TIMP1 to mitigate LPS-induced ALI.

Interferon-stimulated gene 15 (*Isg15*), which encodes the ubiquitin-like protein ISG15, which is primarily induced by type I interferons, is an essential player in regulating host signaling pathways such as damage repair responses and immune responses. ISG15 can be induced by various pathogenic stimuli such as viral and bacterial infections, lipopolysaccharide (LPS), retinoic acid, or certain genotoxic stressors [51]. In accordance with our findings, previous studies have observed increased levels of ISG15 conjugates in macrophages in response to LPS treatment [52]. Moreover, research has found that systemic *ISG* (*MX1, ISG15, IFIT1,* and *IFIT3*) expression within the first days of ARDS onset is associated with disease severity and prognosis. This response should be considered along with other identified genetic, environmental, and complex demographic factors as the cause of heterogeneity in ARDS prognosis [53]. Nevertheless, no data has been reported on the association between ISG15 and AST in the literature.

Since the excessive recruitment of leukocytes appears to be a central contributor to the pathogenesis of ALI, the elevation of proinflammatory cytokines and chemokines is considered the most important factor [54]. Similarly, we found that the expression of chemokines such as *Ccl7, Cxcl5,* and *Cxcl13* were increased after LPS instillation but decreased after AST treatment. Previous reports have documented an increased level of CCL7 in a mouse model of acute LPS-induced lung inflammation [55]. Moreover, the expression of CXCL5 is also rapidly induced in ALI murine models after LPS administration [56]. However, no data has on CXCL13 expression in ALI models has been reported. Therefore, we report for the first time the induction of CXCL13 after LPS administration, which provides insights into the role of CXCL13 in the pathogenesis of ALI. Furthermore, the observation of decreased expression of *Ccl7*, *Cxcl5*, and *Cxcl13* may hint at the anti-inflammatory properties of AST.

Although the roles of other DEGs (*Ly6i, Gbp11,* and *Tgtp1*) have been described in many other diseases in detail, their regulatory mechanisms in ALI-/ ARDS- are not fully understood. Our results show that these DEGs are overexpressed to varying degrees in the LPS group and that AST can effectively prevent this overexpression. Further studies on the roles of these three genes in ALI initiation and progression are need. To determine the functional mechanisms of these 11 DEGs, GO enrichment analyses were further conducted. According to the results, 61 terms in biological process category, 18 in cellular component category and 15 in the molecular function category were enriched. The 5 most significantly enriched terms in the BP category were associated with chemokines and neutrophils, indicating the dominant role of neutrophils and related chemokines in the pathogenesis and progression of LPS-induced ALI. In ALI, the excessive recruitment of inflammatory cells and their mediators results in injury to endothelial and epithelial barriers [54]. Thus, agents such as AST, which can exert robust anti-inflammatory effects, may provide potential treatment prospects.

Despite this, several limitations of the current study need to be addressed. First, our research did not use the ALI mouse models induced by other agents; thus, it did not address heterogeneity of ALI initiation. Second, given that findings in animal models of LPS-induced lung injury may depend on the time point at which samples are obtained and physiological data are captured, the dynamic changes in LPS-induced ALI models may have been ignored to a certain extent [57]. Finally, in-depth research into the underlying mechanisms using knockout-gene mice for each differentially expressed gene will help further our understanding of the role of AST in ameliorating ALI/ARDS.

In conclusion, many genes were dysregulated in ALI/ARDS. We not only identified genes that consistently differed in expression in the LPS group compared to the control group but also revealed that AST can alleviate the abnormal expression of these genes and thus confer a certain therapeutic effect against ALI/ARDS, suggesting the potential for AST to become a novel treatment for ALI/ARDS.

## MATERIALS AND METHODS

### Microarray data meta-analysis

To identify the genes related to LPS-induced acute lung injury in mice, five datasets (GSE102016, GSE2411, GSE16409, GSE104214, and GSE18341) were obtained from GEO (Gene Expression Omnibus, http://www.ncbi.nlm.nih.gov/geo) [58–62]. LPS and control treatments were used in this study. The detailed information (experimental design, transcriptome analysis, array information, data processing, and platform ID) for these datasets can be obtained from the GEO repository, and this information is partly summarized in Table 1 and is described in more detail in Supplementary Table 1.

Then, we conducted a microarray meta-analysis using NetworkAnalyst 3.0 (https://www.networkanalyst.ca) [20]. NetworkAnalyst is a visual analytics platform for comprehensive gene expression profiling and meta-analysis. All gene probes were converted to a common Entrez ID using the gene/probe conversion tool in NetworkAnalyst. Following quantile normalization, all datasets were preprocessed through a log2 transformation and variance stabilizing normalization (VSN). Each dataset was visualized in box plots to ensure an identical distribution among the samples. Differential expression analysis was performed independently for each dataset using NetworkAnalyst, with an FDR of 0.05 and a significance of p < 0.05. The moderated t-test was based on the Limma algorithm. For the meta-analysis, we used Fisher’s method, the fixed-effect model, and vote counting (combined p < 0.05 or vote counts ≥ 3 were considered significant) to identify the differentially expressed genes (DEGs) and we selected the common DEGs identified by these three methods as the final DEGs.

### Animal materials

Male C57BL/6J mice (18–24g, 6~8-weeks-old) were purchased from Beijing Vital River Laboratory Animal Technology Co., Ltd. (Beijing, China). The mice were housed 5 per cage under a 12h light/dark cycle in a laboratory at 23 ± 2 °C and 50% humidity. All experiment protocols conformed to the guidelines of the China Council on Animal Care and Use. These animal studies were approved by the Institutional Animal Research Committee of Union Hospital.

The mice were randomly allocated into three groups: (1) the control group (n=10), which was exposed to PBS alone and received an intraperitoneal injection of sterile saline; (2) the LPS group (n=10), which was exposed to PBS containing 0.5 mg/mL LPS; and (3) the AST group (n=10), which was intraperitoneally injected with AST (10 mg/ml, dissolved in PBS) at a dosage of 50mg/kg body weight every day before one week of exposure to LPS to evaluate its preventive and protective effects [19, 63], and intraperitoneally injected with 100mg/kg AST (20 mg/ml, dissolved in PBS) 24 hours after LPS exposure in order to confirm the therapeutic effect of AST [16]. AST was obtained from Sigma-Aldrich (St Louis, MO, USA). For acute LPS exposure, mice were exposed to an aerosol of phosphate buffer saline (PBS) alone or PBS containing 0.5 mg/mL LPS for 2h, in a custom-built cuboidal chamber. The LPS solution was aerosolized with a constant output ultrasonic nebulizer (model: 402B, Yuwell, China) at a flow rate of 35ml/h. LPS was purchased from Sigma–Aldrich (extracted from Escherichia coli O55: B5, L2880). The chamber was 20 cm long, 15 cm wide and 15 cm high.

### RNA-seq library and sequencing

Total RNA was extracted from mouse lung tissue samples with TRIzol® Reagent (Invitrogen, CA) following the manufacturer's protocol. The concentration and purity of the RNA were measured by a Nanodrop2000 spectrophotometer (NanoDrop Technologies, Technologies, Wilmington, DE, USA), the RNA integrity was detected by agarose gel electrophoresis, and the RIN was determined using an Agilent2100 Bioanalyzer (Agilent Technologies, Santa Clara, CA, USA). The construction of a single library required a total of 5μg RNA with a concentration of ≥200ng/μL and an OD 260/280 ratio between

1.8 and 2.2. Then, oligo (dT) magnetic beads were subjected to capture mRNAs that contained poly-A tails from the total RNA. The resulting mRNAs were subsequently randomly broken into small fragments of approximately 200 bp by adding fragmentation buffer. The mRNA fragments functioned as the templates for double-stranded cDNA (dscDNA) synthesis using the SuperScript double-stranded cDNA synthesis kit (Invitrogen, CA, USA). Under the action of reverse transcriptase, a strand of cDNA was synthesized by using random primers with mRNA as a template, which was followed by two-strand synthesis to form a stable double-stranded structure. Since there was a cohesive terminus in the double-stranded cDNA structure, End Repair Mix was added to patch it into a blunt end, and an A base was added at the 3 'end to connect the y-shaped adaptor. To purify and enrich the dscDNA, 15 cycles of PCR were performed, and clean DNA beads were used to screen 200-300 bp bands. After quantification by TBS380 (Picogreen, Invitrogen, CA, USA), high-throughput sequencing of the resulting libraries was performed on the Illumina HiSeq xten/NovaSeq 6000 sequencing platform (San Diego, CA, USA), and the sequencing read length was paired-end (PE) 150.

### Quality control of raw data

To ensure the accuracy of the subsequent biological information analysis, the raw sequencing data generated from RNA-Seq was firstly filtered to obtain high-quality sequencing data (clean data) to ensure the smooth progress of the subsequent analysis. Quality control of the raw reads was performed using SeqPrep (https://github.com/jstjohn/SeqPrep) and Sickle (https://github.com/najoshi/sickle). The processes were as follows. The first step was to remove the adapter from the reads and the reads that did not insert the fragment due to the self-connection of the adapter. Second, bases with a low quality (quality value less than 20) at the end of the sequence (3' end) were trimmed. If there was still a quality value of less than 10 for the remaining sequence, the whole sequence was discarded; otherwise, it was retained. Third, reads with N ratios over 10% and sequences with lengths less than 20 bp after quality trimming were also removed. Finally, the error rate (%), Q20 and Q30 values, GC-content (%), and sequence duplication levels of the generated clean reads were assessed [64].

### Data process and DEG analysis

After filtering the raw data, the clean data were aligned to the mouse reference genome GRCm38 by ‘Bowtie2’ software [65]. Then, read summarization was calculated by the 'feature count' tool. Differently expressed genes (DEGs) between the LPS samples and control samples were identified by t-test using the 'DEseq2’ R package, as were DEGs between the AST samples and LPS samples [66]. The raw P-value was adjusted to the false discovery rate by the Benjamini method, and a false discovery rate (FDR) ≤ 0.05 and |log2FC|≥ 1 was chosen as the threshold.

### Functional enrichment analyses of DEGs

Based on the hypergeometric distribution algorithm, GO (Gene Ontology, http://www.geneontology.org/) biological process (BP), molecular function (MF) and cell component (CC) pathway enrichment analyses were performed using the ‘clusterprofler’ R package [21]. A P-value ≤ 0.05 was set as the cutoff criterion.

### RNA isolation and qRT-PCR validation

To validate the combined findings from RNA-seq and microarray meta-analysis, the expression of 11 core DEGs in the three groups was confirmed. RNAiso Plus reagent (Takara, Tokyo, Japan) was employed to extract total RNA from mouse lung tissues from each group, and reverse transcription was performed to obtain cDNA using PrimeScript™RT Master Mix (Takara, Tokyo, Japan) along with the gDNA Eraser kit (Takara, Tokyo, Japan). Relative mRNA expression levels were determined using RT-PCR performed on Bio-Rad CFX Maestro (Bio-Rad, USA) with TB Green® Premix Ex Taq™ II (Takara, Tokyo, Japan). All the above experimental steps were performed according to the manufacturer’s instructions for the corresponding kit. Glyceraldehyde-3-phosphate dehydrogenase (GAPDH) was selected as the reference, and the primer sequences are presented in Supplementary Table 6. qRT-PCR was performed under the following conditions: 95 °C for 3 min, followed by 40 cycles at 95 °C for 30 s, 56 °C for 30 s, and 72 °C for 30 s. Each analysis was implemented in triplicate, and the relative expression levels of the target genes were calculated by employing the 2-ΔΔCt method [67].

### Statistical analysis

INMEX and NetworkAnalyst were applied for the network-based microarray meta-analysis. For qPCR data, statistical analysis of differences between groups was achieved by one-way ANOVA using Prism 7 software (GraphPad Software Inc., San Diego, CA, USA). A two-tailed test was used for all data, and differences with a P-value <0.05 were considered significant.

## Supplementary Material

Supplementary Materials and Methods

Supplementary Figures

Supplementary Table 1

Supplementary Table 2

Supplementary Table 3

Supplementary Tables 4, 5 and 6
