# Supplementary material for: Identification of robust genetic signatures associated with lipopolysaccharide-induced acute lung injury onset and astaxanthin therapeutic effects by integrative analysis of RNA sequencing data and GEO datasets
Source: Aging (Albany NY). 2020 Sep 23;12(18):18716–40. doi: 10.18632/aging.104042 (PMC7585091; doi:10.18632/aging.104042)
Supplement: Supplementary Materials and Methods [file aging-12-104042-s003..pdf]

SUPPLEMENTARY MATERIALS AND METHODS

Microarray data meta-analysis

First of all, we used the search formula of LPS[All Fields] AND ("lung"[MeSH Terms] OR lung[All Fields]) AND ("Mus musculus"[Organism] AND "Expression profiling by array"[Filter]) to obtain 62 results in GEO DataSets. By eliminating datasets of miRNA sequencings, datasets not related to acute lung injury, and datasets that only researching on RNA sequencing of specific cells such as macrophages and type II alveolar epithelial cells, there were 8 articles remained (GSE71648, GSE104214, GSE102016, GSE38014, GSE18341, GSE16409, GSE11662 and GSE2411). Continuing to check the specific description of the sample in articles, we found some datasets were grouped with sample of n<3 and some mainly studied ALI or ARDS induced by excessive ventilation or non-LPS chemicals. In the end, there were 5 datasets (GSE102016, GSE2411, GSE16409, GSE104214 and GSE18341) that met the requirements of integrated analysis.

We conducted a microarray meta-analysis using NetworkAnalyst 3.0 combined three well-established meta-analysis approaches --Fisher's method, Fixed effect model, and Vote counting. The features and main characteristics are given below (https://www.networkanalyst.ca). (1) Fisher's method ($-2*\sum Log(p)$) is known as a 'weight-free' method and combines p values from multiple studies for information integration. (2) Effect size is the difference between two group means divided by standard deviation, which are considered combinable and comparable across different studies. In the fixed effects models (FEM), the estimated effect size in each study is assumed to come from an underlying true effect size plus measurement error. (3) Vote counting is the simplest method in meta-analysis. Differentially expressed gene is first selected based on a threshold to obtain a list of DE genes for each study. The vote for each gene can then be calculated as the total number of times it occurred in all DE lists. The final DE genes can be selected based on the minimal number of votes set by the user.

Immunohistochemistry

After the mice were sacrificed, the lung tissues were collected. Immediately, the tissue was fixed in 4% paraformaldehyde for 24 hours and embedded in paraffin. The embedded tissue was sliced into 5 μm sections for staining. After the tissue sections were deparaffinized and rehydrated, they were heated in citrate buffer at 121 °C for 30 minutes to restore antigen activity. The sections were then incubated with 0.3% hydrogen peroxide in methanol for 30 minutes to inhibit endogenous peroxidase activity. After blocking nonspecific reactions with 10% normal bovine serum, the sections were incubated with rabbit polyclonal antibodies specific for Ccl7 (1:100, Abcam), Saa3 (1:50, ab231680, Abcam), Ly6i (1:2000, Abcam), Saa1 (1:100-1:200, Thermo), lrf7 (1:100, Thermo), Timp1 (1:100, Thermo), Isg15 (1:100, Thermo) and Cxcl13 (1:1000, Abcam). The treated samples were placed at 4 °C for 12 hours. The sections were then washed with PBS and incubated with horseradish peroxidase-conjugated secondary antibodies at 37 °C for 2 hours. The stained sections were imaged under an inverted phase contrast microscope.
