# Supplementary material for: Identification of robust genetic signatures associated with lipopolysaccharide-induced acute lung injury onset and astaxanthin therapeutic effects by integrative analysis of RNA sequencing data and GEO datasets
Source: Aging (Albany NY). 2020 Sep 23;12(18):18716–40. doi: 10.18632/aging.104042 (PMC7585091; doi:10.18632/aging.104042)
Supplement: Supplementary Figures [file aging-12-104042-s005..pdf]

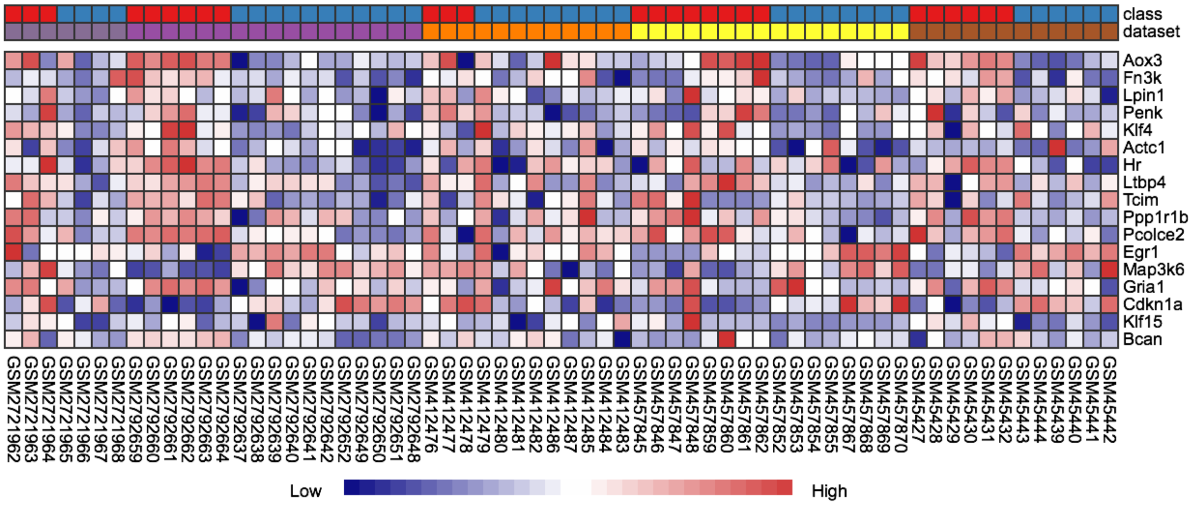

Supplementary Figure 1. Heat map of the 17 overlapping downregulated genes identified by both meta-analysis of published microarrays and our RNA sequencing experiments (control group vs. LPS group).

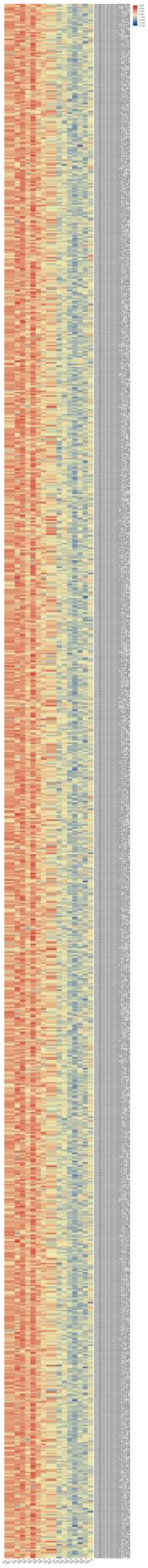

Supplementray Figure 2-1. Heat map of the 989 significantly upregulated genes in the LPS group compared to the control group identified by RNA sequencing.

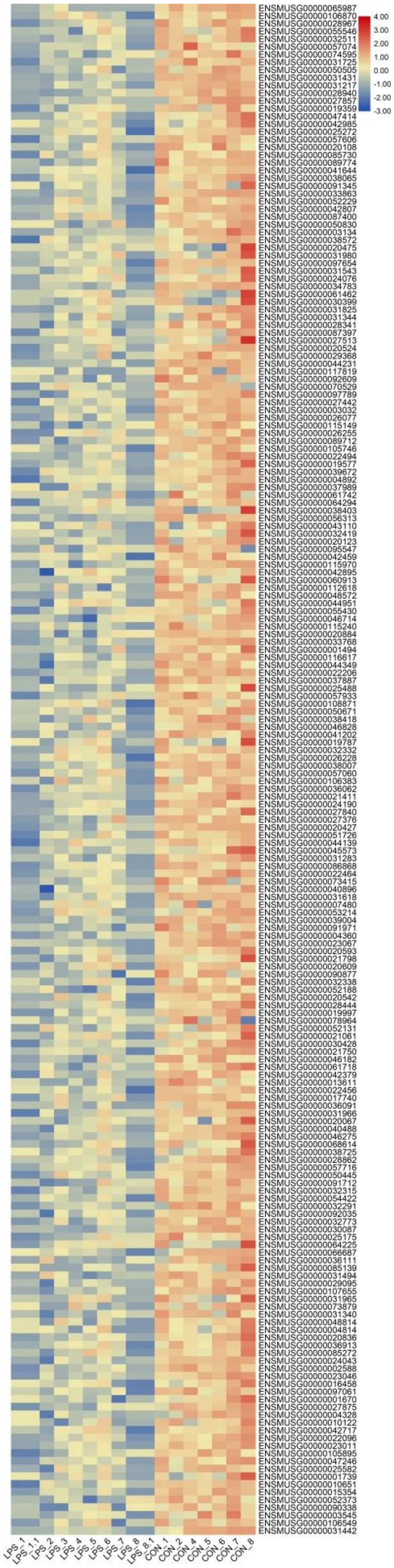

Supplementary Figure 2-2. Heat map of the 198 significantly downregulated genes in the LPS group compared to the control group identified by RNA sequencing.

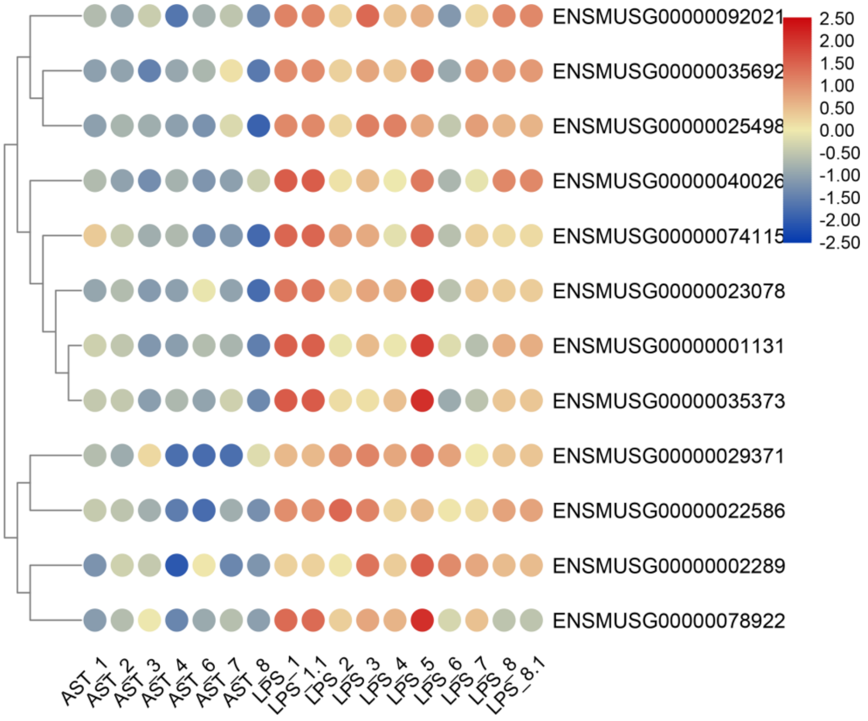

Supplementary Figure 3-1. Heat map of the 9 significantly upregulated genes in the AST group compared to the LPS group identified by RNA sequencing.

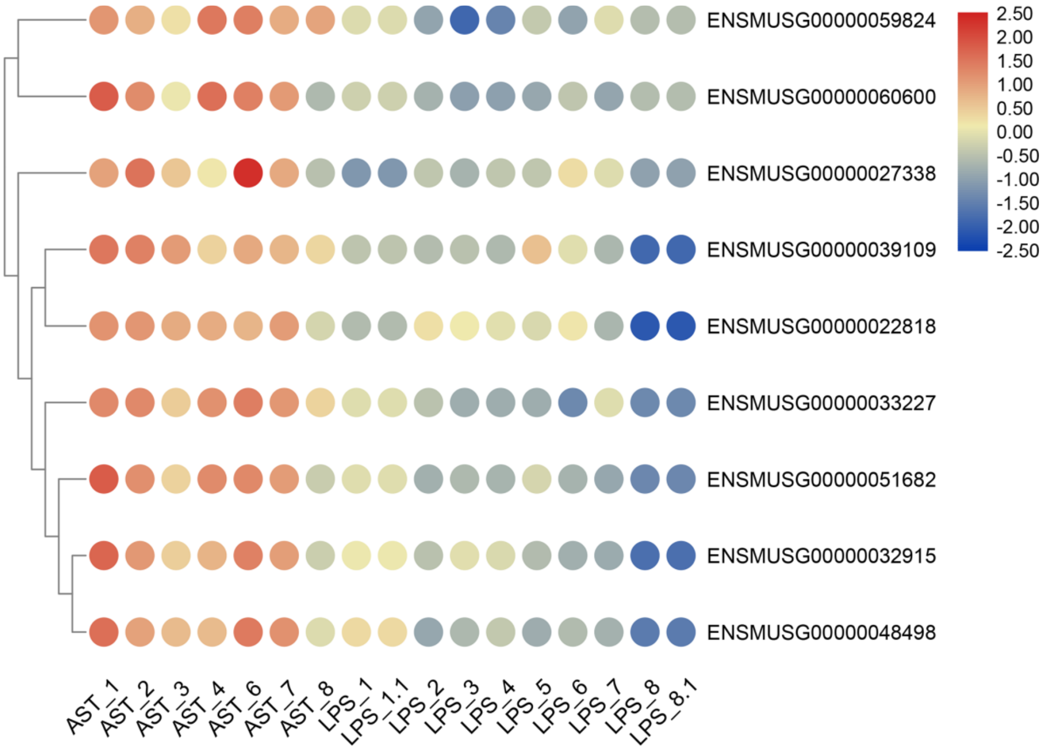

Supplementary Figure 3-2. Heat map of the 12 significantly downregulated genes in the AST group compared to the LPS group identified RNA sequencing.

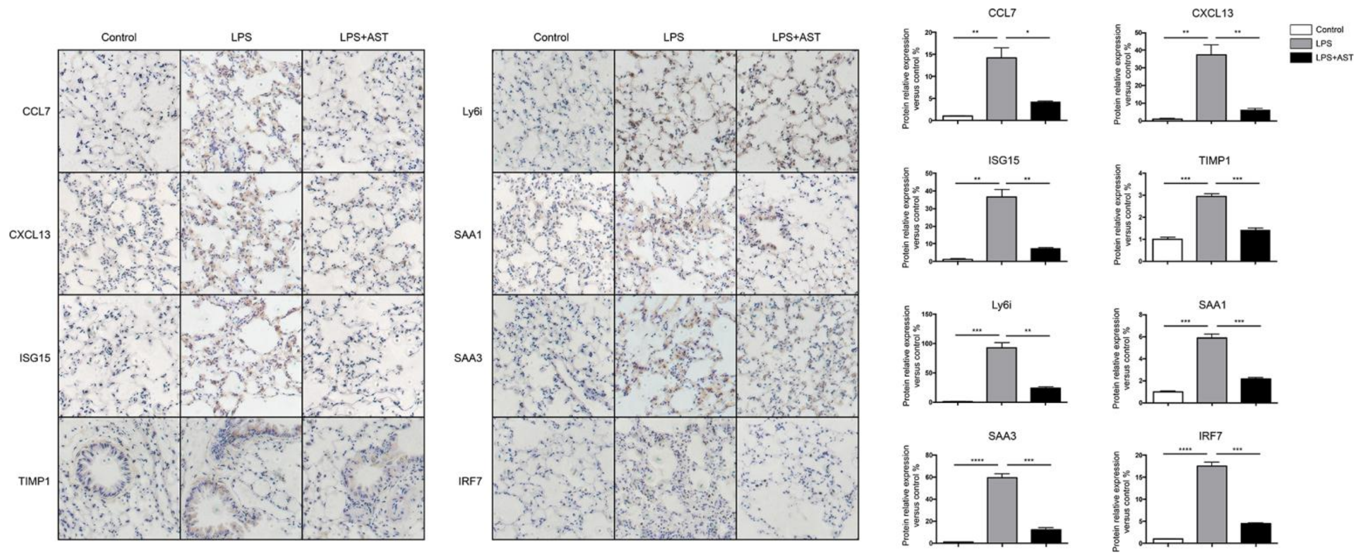

Supplementary Figure 4. Expression of the 8 core genes (*Ccl7, Saa3, Ly6i, Saa1, Irf7, Timp1, Isg15* and *Cxcl13*) determined by immunohistochemistry. Statistical analysis of the differences between groups was achieved with one-way ANOVA using Prism 7 software. ****$p < 0.0001$, ***$p < 0.001$, **$p < 0.01$, and *$p < 0.05$ were considered statistically significant.
