# Supplementary material for: Identification of robust genetic signatures associated with lipopolysaccharide-induced acute lung injury onset and astaxanthin therapeutic effects by integrative analysis of RNA sequencing data and GEO datasets
Source: Aging (Albany NY). 2020 Sep 23;12(18):18716–40. doi: 10.18632/aging.104042 (PMC7585091; doi:10.18632/aging.104042)
Supplement: Supplementary Table 1 [file aging-12-104042-s001..docx]

| **Table S1. Characteristics of studies composing the LPS-induced ALI gene expression compendium.** | | | |
| --- | --- | --- | --- |
| **GEO Dataset** | **Samples used in this study** | | **Seq Platform** |
| GSE102016 | GSM2721962 | Mouse lung control, biological rep 1 | Affymetrix Mouse Gene 1.1 ST Array |
|  | GSM2721963 | Mouse lung control, biological rep 2 |  |
|  | GSM2721964 | Mouse lung control, biological rep 3 |  |
|  | GSM2721965 | Mouse lung LPS, biological rep 1 |  |
|  | GSM2721966 | Mouse lung LPS, biological rep 2 |  |
|  | GSM2721967 | Mouse lung LPS, biological rep 3 |  |
|  | GSM2721968 | Mouse lung LPS, biological rep 4 |  |
| GSE104214 | GSM2792637 | C57BL/6 8H WT1 | Agilent-028005 SurePrint G3 Mouse GE 8x60K Microarray |
|  | GSM2792638 | C57BL/6 8H WT2 |  |
|  | GSM2792639 | C57BL/6 8H WT3 |  |
|  | GSM2792640 | C57BL/6 8H WT4 |  |
|  | GSM2792641 | C57BL/6 8H WT5 |  |
|  | GSM2792642 | C57BL/6 8H WT6 |  |
|  | GSM2792648 | C57BL/6 24H WT1 |  |
|  | GSM2792649 | C57BL/6 24H WT2 |  |
|  | GSM2792650 | C57BL/6 24H WT3 |  |
|  | GSM2792651 | C57BL/6 24H WT4 |  |
|  | GSM2792652 | C57BL/6 24H WT5 |  |
|  | GSM2792659 | C57BL/6 CTRL WT1 |  |
|  | GSM2792660 | C57BL/6 CTRL WT2 |  |
|  | GSM2792661 | C57BL/6 CTRL WT3 |  |
|  | GSM2792662 | C57BL/6 CTRL WT4 |  |
|  | GSM2792663 | C57BL/6 CTRL WT5 |  |
|  | GSM2792664 | C57BL/6 CTRL WT6 |  |
| GSE2411 | GSM45427 | Control 1 | Affymetrix Mouse Expression 430A Array |
|  | GSM45428 | Control 2 |  |
|  | GSM45429 | Control 3 |  |
|  | GSM45430 | Control 4 |  |
|  | GSM45431 | Control 5 |  |
|  | GSM45432 | Control 6 |  |
|  | GSM45439 | LPS 1 |  |
|  | GSM45440 | LPS 2 |  |
|  | GSM45441 | LPS 3 |  |
|  | GSM45442 | LPS 4 |  |
|  | GSM45443 | LPS 5 |  |
|  | GSM45444 | LPS 6 |  |
| GSE16409 | GSM412476 | Wild-type, Control, Replicate 1 | Duke-GE/Amersham CodeLink UniSet Mouse 20K I Bioarray |
|  | GSM412477 | Wild-type, Control, Replicate 2 |  |
|  | GSM412478 | Wild-type, Control, Replicate 3 |  |
|  | GSM412479 | Wild-type, 1.5 hours post LPS, Replicate 1 |  |
|  | GSM412480 | Wild-type, 1.5 hours post LPS, Replicate 2 |  |
|  | GSM412481 | Wild-type, 1.5 hours post LPS, Replicate 3 |  |
|  | GSM412482 | Wild-type, 6 hours post LPS, Replicate 1 |  |
|  | GSM412483 | Wild-type, 6 hours post LPS, Replicate 2 |  |
|  | GSM412484 | Wild-type, 6 hours post LPS, Replicate 3 |  |
|  | GSM412485 | Wild-type, 12 hours post LPS, Replicate 1 |  |
|  | GSM412486 | Wild-type, 12 hours post LPS, Replicate 2 |  |
|  | GSM412487 | Wild-type, 12 hours post LPS, Replicate 3 |  |
| GSE18341 | GSM457845 | lung_21 day old, untreated, spontaneously breathing control_rep1 | Affymetrix Mouse Genome 430 2.0 Array |
|  | GSM457846 | lung_21 day old, untreated, spontaneously breathing control_rep2 |  |
|  | GSM457847 | lung_21 day old, untreated, spontaneously breathing control_rep3 |  |
|  | GSM457848 | lung_21 day old, untreated, spontaneously breathing control_rep4 |  |
|  | GSM457852 | lung_21 day old, treated with inhaled LPS, then spontaneous breathing for 2 hr_rep1 |  |
|  | GSM457853 | lung_21 day old, treated with inhaled LPS, then spontaneous breathing for 2 hr_rep2 |  |
|  | GSM457854 | lung_21 day old, treated with inhaled LPS, then spontaneous breathing for 2 hr_rep3 |  |
|  | GSM457855 | lung_21 day old, treated with inhaled LPS, then spontaneous breathing for 2 hr_rep4 |  |
|  | GSM457859 | lung_16 week old, untreated, spontaneously breathing control_rep1 |  |
|  | GSM457860 | lung_16 week old, untreated, spontaneously breathing control_rep2 |  |
|  | GSM457861 | lung_16 week old, untreated, spontaneously breathing control_rep3 |  |
|  | GSM457862 | lung_16 week old, untreated, spontaneously breathing control_rep4 |  |
|  | GSM457867 | lung_16 week old, treated with inhaled LPS, then spontaneous breathing for 2 hr_rep1 |  |
|  | GSM457868 | lung_16 week old, treated with inhaled LPS, then spontaneous breathing for 2 hr_rep2 |  |
|  | GSM457869 | lung_16 week old, treated with inhaled LPS, then spontaneous breathing for 2 hr_rep3 |  |
|  | GSM457870 | lung_16 week old, treated with inhaled LPS, then spontaneous breathing for 2 hr_rep4 |  |
