# Supplementary figures and images for: Identification of robust genetic signatures associated with lipopolysaccharide-induced acute lung injury onset and astaxanthin therapeutic effects by integrative analysis of RNA sequencing data and GEO datasets
Source: Aging (Albany NY). 2020 Sep 23;12(18):18716–40. doi: 10.18632/aging.104042 (PMC7585091; doi:10.18632/aging.104042)

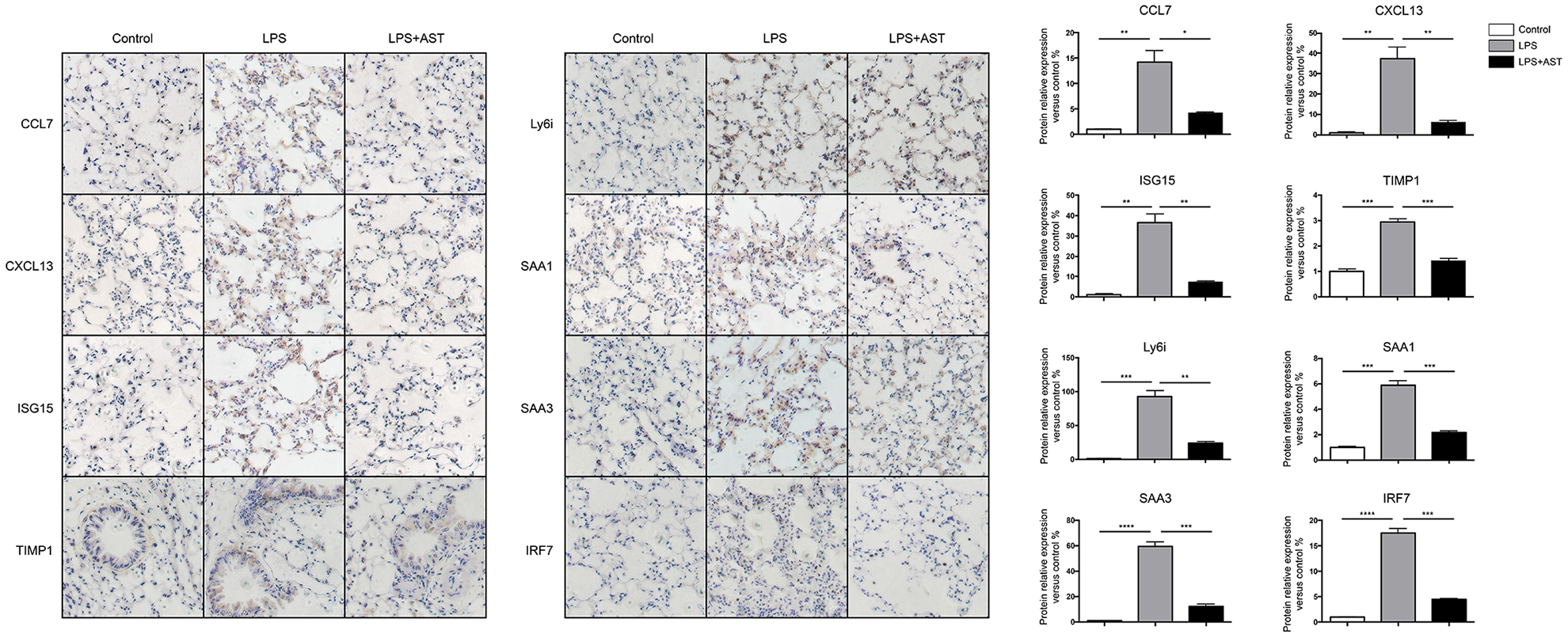

Supplement: Supplementary Table 2 [file aging-12-104042-s006..tif]
