# Supplementary material for: Identification of robust genetic signatures associated with lipopolysaccharide-induced acute lung injury onset and astaxanthin therapeutic effects by integrative analysis of RNA sequencing data and GEO datasets
Source: Aging (Albany NY). 2020 Sep 23;12(18):18716–40. doi: 10.18632/aging.104042 (PMC7585091; doi:10.18632/aging.104042)
Supplement: Supplementary Table 3 [file aging-12-104042-s002..docx]

**Table S3. DEGs between LPS and control samples.**

| **Gene** | **sampleA** | **sampleB** | **baseMeanA** | **baseMeanB** | **baseMean** | **log2FoldChange** | **lfcSE** | **stat** | **pvalue** | **padj** |
| --- | --- | --- | --- | --- | --- | --- | --- | --- | --- | --- |
| *ENSMUSG00000020609* | LPS | CON | 5.485287052 | 14.23515695 | 9.088174658 | -1.000799074 | 0.378507374 | -2.644067575 | 0.00819163 | 0.035478203 |
| *ENSMUSG00000020475* | LPS | CON | 8.982648458 | 25.33833453 | 15.71734272 | -1.000849249 | 0.408877882 | -2.447795035 | 0.014373339 | 0.054198856 |
| *ENSMUSG00000106549* | LPS | CON | 5.32224072 | 12.75193064 | 8.381524804 | -1.001118855 | 0.334932891 | -2.989013268 | 0.0027988 | 0.015260445 |
| *ENSMUSG00000057060* | LPS | CON | 6.106423322 | 15.50266774 | 9.975465143 | -1.003770407 | 0.369042258 | -2.719933521 | 0.006529504 | 0.02975626 |
| *ENSMUSG00000044951* | LPS | CON | 12.5238138 | 33.41180914 | 21.12475306 | -1.005455914 | 0.391076185 | -2.570997549 | 0.010140604 | 0.041883492 |
| *ENSMUSG00000078964* | LPS | CON | 4.176929767 | 12.33790335 | 7.537330654 | -1.006296779 | 0.415352728 | -2.422752308 | 0.015403425 | 0.056976183 |
| *ENSMUSG00000108871* | LPS | CON | 7.210838192 | 18.48509496 | 11.85317921 | -1.006871712 | 0.369546067 | -2.724617586 | 0.0064376 | 0.029432507 |
| *ENSMUSG00000042379* | LPS | CON | 103.5731569 | 219.5313557 | 151.3206505 | -1.008501213 | 0.220605855 | -4.571507023 | 4.84E-06 | 6.49E-05 |
| *ENSMUSG00000097061* | LPS | CON | 18.01648852 | 38.53458978 | 26.46511845 | -1.009197198 | 0.22680613 | -4.449602826 | 8.60E-06 | 0.00010741 |
| *ENSMUSG00000042895* | LPS | CON | 9.469777805 | 23.08098686 | 15.0743933 | -1.014182846 | 0.341972934 | -2.965681625 | 0.00302013 | 0.016242311 |
| *ENSMUSG00000074595* | LPS | CON | 7.780347613 | 17.52431144 | 11.79256801 | -1.015077678 | 0.321835077 | -3.154030591 | 0.001610322 | 0.00969069 |
| *ENSMUSG00000053214* | LPS | CON | 9.592493379 | 22.29640465 | 14.82351567 | -1.017329201 | 0.304648075 | -3.33935871 | 0.000839721 | 0.005709425 |
| *ENSMUSG00000001739* | LPS | CON | 41.26395276 | 100.3032817 | 65.57426467 | -1.019103532 | 0.35033038 | -2.908978472 | 0.003626118 | 0.018756548 |
| *ENSMUSG00000052373* | LPS | CON | 24.23296906 | 54.52992079 | 36.70818448 | -1.019444874 | 0.301572474 | -3.380430777 | 0.000723723 | 0.005023629 |
| *ENSMUSG00000091345* | LPS | CON | 3.670376809 | 9.810425815 | 6.198632282 | -1.023255535 | 0.386993012 | -2.644118891 | 0.008190388 | 0.035478203 |
| *ENSMUSG00000032332* | LPS | CON | 234.607527 | 504.3702585 | 345.6862988 | -1.023961602 | 0.220888212 | -4.635655259 | 3.56E-06 | 4.96E-05 |
| *ENSMUSG00000004814* | LPS | CON | 2.248173097 | 7.466674459 | 4.396967775 | -1.025114167 | 0.423514129 | -2.420495793 | 0.015499359 | 0.057255977 |
| *ENSMUSG00000046828* | LPS | CON | 27.12686358 | 58.15285561 | 39.90227207 | -1.029398479 | 0.217275094 | -4.737765664 | 2.16E-06 | 3.18E-05 |
| *ENSMUSG00000007480* | LPS | CON | 1.679185285 | 5.961697611 | 3.442572713 | -1.030557191 | 0.425555976 | -2.421672466 | 0.015449268 | 0.057120793 |
| *ENSMUSG00000087400* | LPS | CON | 49.32120359 | 107.8684079 | 73.42887593 | -1.030601937 | 0.227701083 | -4.52611785 | 6.01E-06 | 7.85E-05 |
| *ENSMUSG00000064294* | LPS | CON | 530.3396977 | 1145.041535 | 783.4522189 | -1.030747176 | 0.222229884 | -4.638202376 | 3.51E-06 | 4.91E-05 |
| *ENSMUSG00000021411* | LPS | CON | 957.9708909 | 1988.43975 | 1382.281598 | -1.03150953 | 0.124131471 | -8.30981478 | 9.59E-17 | 9.33E-15 |
| *ENSMUSG00000023011* | LPS | CON | 87.96268474 | 184.102486 | 127.5496617 | -1.03161922 | 0.142878961 | -7.220231795 | 5.19E-13 | 2.96E-11 |
| *ENSMUSG00000061742* | LPS | CON | 1.955878896 | 6.176786447 | 3.693899653 | -1.032729269 | 0.417661614 | -2.472645877 | 0.013411697 | 0.05155768 |
| *ENSMUSG00000044139* | LPS | CON | 8.570613044 | 19.49504173 | 13.06890721 | -1.039201603 | 0.280800177 | -3.700858077 | 0.000214872 | 0.001767627 |
| *ENSMUSG00000046714* | LPS | CON | 4.356451288 | 10.53081379 | 6.898835847 | -1.039942437 | 0.358265542 | -2.902714092 | 0.003699442 | 0.018985012 |
| *ENSMUSG00000025175* | LPS | CON | 5.467849916 | 12.89823112 | 8.527418649 | -1.040789815 | 0.322044482 | -3.231820051 | 0.001230045 | 0.007753038 |
| *ENSMUSG00000026228* | LPS | CON | 4.16557518 | 10.80991962 | 6.901481712 | -1.041520425 | 0.348058684 | -2.992370174 | 0.002768204 | 0.015137497 |
| *ENSMUSG00000024190* | LPS | CON | 1825.478569 | 3896.420256 | 2678.219264 | -1.042002547 | 0.183302141 | -5.684617444 | 1.31E-08 | 3.30E-07 |
| *ENSMUSG00000091712* | LPS | CON | 11.41137494 | 25.83543885 | 17.35069537 | -1.042777014 | 0.290021651 | -3.595514369 | 0.000323751 | 0.002529353 |
| *ENSMUSG00000017740* | LPS | CON | 17.33337224 | 38.7814356 | 26.16492774 | -1.044938738 | 0.238293715 | -4.385087276 | 1.16E-05 | 0.000139209 |
| *ENSMUSG00000003134* | LPS | CON | 361.9993467 | 765.2789052 | 528.0556355 | -1.045398674 | 0.155202333 | -6.735714949 | 1.63E-11 | 7.08E-10 |
| *ENSMUSG00000027857* | LPS | CON | 5.251239819 | 12.04117329 | 8.047094776 | -1.053586108 | 0.289233668 | -3.642681412 | 0.000269813 | 0.0021557 |
| *ENSMUSG00000064225* | LPS | CON | 2.830579023 | 11.5581153 | 6.424270432 | -1.056586708 | 0.431813304 | -2.446860017 | 0.01441068 | 0.054284894 |
| *ENSMUSG00000042717* | LPS | CON | 12.60397346 | 30.71058387 | 20.05963657 | -1.056948019 | 0.316672137 | -3.337672923 | 0.000844831 | 0.005732954 |
| *ENSMUSG00000090338* | LPS | CON | 3.031120662 | 8.531953521 | 5.296169487 | -1.06046624 | 0.397105835 | -2.670487682 | 0.007574115 | 0.033401867 |
| *ENSMUSG00000020542* | LPS | CON | 9.89149734 | 24.99256228 | 16.1095829 | -1.061524988 | 0.335702953 | -3.162096066 | 0.001566379 | 0.009474381 |
| *ENSMUSG00000036091* | LPS | CON | 3.211676641 | 8.059548879 | 5.207859327 | -1.063303548 | 0.337051416 | -3.15472209 | 0.001606511 | 0.009679083 |
| *ENSMUSG00000020593* | LPS | CON | 217.4365198 | 473.9528208 | 323.060879 | -1.065284525 | 0.188142915 | -5.662102787 | 1.50E-08 | 3.71E-07 |
| *ENSMUSG00000038065* | LPS | CON | 258.9440336 | 559.9921711 | 382.9050314 | -1.06858488 | 0.164539321 | -6.494404341 | 8.34E-11 | 3.20E-09 |
| *ENSMUSG00000025582* | LPS | CON | 121.9005324 | 271.0272495 | 183.3056512 | -1.068900301 | 0.219864275 | -4.861637036 | 1.16E-06 | 1.85E-05 |
| *ENSMUSG00000045573* | LPS | CON | 7.776569316 | 18.51918653 | 12.19999993 | -1.069933687 | 0.321227055 | -3.330770779 | 0.000866059 | 0.005843913 |
| *ENSMUSG00000027875* | LPS | CON | 152.2114984 | 342.331459 | 230.496188 | -1.071381373 | 0.242901189 | -4.410770393 | 1.03E-05 | 0.000125728 |
| *ENSMUSG00000003032* | LPS | CON | 863.7742146 | 1841.765123 | 1266.476354 | -1.071414984 | 0.116695821 | -9.181262698 | 4.26E-20 | 6.87E-18 |
| *ENSMUSG00000038007* | LPS | CON | 2640.597559 | 5607.880677 | 3862.420019 | -1.075545054 | 0.084790375 | -12.68475408 | 7.18E-37 | 8.11E-34 |
| *ENSMUSG00000031344* | LPS | CON | 5.128414472 | 13.7086746 | 8.661462759 | -1.075635633 | 0.359254135 | -2.994080035 | 0.002752737 | 0.01507727 |
| *ENSMUSG00000036111* | LPS | CON | 5.607529024 | 13.61499299 | 8.904720068 | -1.078064433 | 0.33782177 | -3.191222495 | 0.001416721 | 0.008702864 |
| *ENSMUSG00000019997* | LPS | CON | 884.1314901 | 1913.371258 | 1307.9361 | -1.078538081 | 0.1510703 | -7.139312515 | 9.38E-13 | 5.16E-11 |
| *ENSMUSG00000027513* | LPS | CON | 1.211341933 | 335.0614735 | 4.087020585 | -1.081470255 | 0.42429523 | -2.548862628 | 0.010807486 | 0.043943165 |
| *ENSMUSG00000021061* | LPS | CON | 19.5365914 | 48.38442767 | 31.41511222 | -1.082569055 | 0.322040793 | -3.361589832 | 0.000774951 | 0.005311712 |
| *ENSMUSG00000092609* | LPS | CON | 10.38159385 | 24.38738529 | 16.14868444 | -1.085051375 | 0.286764196 | -3.783775628 | 0.000154467 | 0.001334249 |
| *ENSMUSG00000039672* | LPS | CON | 114.5949612 | 261.3493096 | 175.0232223 | -1.088318386 | 0.238855131 | -4.556395261 | 5.20E-06 | 6.92E-05 |
| *ENSMUSG00000024043* | LPS | CON | 155.6368533 | 339.1253712 | 231.1909489 | -1.089863084 | 0.147805726 | -7.373618838 | 1.66E-13 | 1.01E-11 |
| *ENSMUSG00000105895* | LPS | CON | 4.912983268 | 12.20495158 | 7.915558453 | -1.090560955 | 0.330297225 | -3.301756336 | 0.000960815 | 0.006362888 |
| *ENSMUSG00000044231* | LPS | CON | 38.37660996 | 83.41530604 | 56.92195541 | -1.093963817 | 0.138105921 | -7.92119419 | 2.35E-15 | 1.84E-13 |
| *ENSMUSG00000089774* | LPS | CON | 77.49142388 | 170.3952955 | 115.7459593 | -1.094328455 | 0.165988431 | -6.592799557 | 4.32E-11 | 1.74E-09 |
| *ENSMUSG00000046275* | LPS | CON | 103.5339951 | 226.2314416 | 154.0564731 | -1.099453276 | 0.137506744 | -7.995631634 | 1.29E-15 | 1.05E-13 |
| *ENSMUSG00000052131* | LPS | CON | 4.90649518 | 12.91020355 | 8.202139802 | -1.106956772 | 0.350856767 | -3.15501047 | 0.001604923 | 0.009672963 |
| *ENSMUSG00000027376* | LPS | CON | 4.747660109 | 12.23082851 | 7.828964746 | -1.113588626 | 0.340639976 | -3.269107285 | 0.001078874 | 0.006990178 |
| *ENSMUSG00000010122* | LPS | CON | 5.227531719 | 13.89240573 | 8.795421018 | -1.114287797 | 0.354479624 | -3.143446674 | 0.001669708 | 0.009992281 |
| *ENSMUSG00000043110* | LPS | CON | 52.50770383 | 162.1024592 | 97.63495606 | -1.117886234 | 0.402013076 | -2.78072108 | 0.005423832 | 0.025649572 |
| *ENSMUSG00000025272* | LPS | CON | 10.78873104 | 27.76082177 | 17.77723899 | -1.119283186 | 0.314728676 | -3.556343198 | 0.000376053 | 0.002871706 |
| *ENSMUSG00000042459* | LPS | CON | 3.400718289 | 9.589095735 | 5.948873708 | -1.121965305 | 0.358759222 | -3.127349028 | 0.001763904 | 0.01046894 |
| *ENSMUSG00000019359* | LPS | CON | 40.69300261 | 100.625585 | 65.37112475 | -1.123452407 | 0.299583315 | -3.750049994 | 0.000176799 | 0.001501863 |
| *ENSMUSG00000107655* | LPS | CON | 72.67346269 | 162.5667858 | 109.6883604 | -1.127486237 | 0.152550729 | -7.390893796 | 1.46E-13 | 9.15E-12 |
| *ENSMUSG00000047246* | LPS | CON | 29.24002592 | 68.49083651 | 45.4021244 | -1.129229517 | 0.229787317 | -4.9142378 | 8.91E-07 | 1.46E-05 |
| *ENSMUSG00000087397* | LPS | CON | 2.495660144 | 7.680466203 | 4.630580286 | -1.129490002 | 0.389650742 | -2.898724118 | 0.003746844 | 0.019164372 |
| *ENSMUSG00000073415* | LPS | CON | 34.00919574 | 80.91752896 | 53.32439177 | -1.133789151 | 0.249080773 | -4.551893497 | 5.32E-06 | 7.04E-05 |
| *ENSMUSG00000029095* | LPS | CON | 19.00027234 | 43.652386 | 29.15114267 | -1.134569125 | 0.188966419 | -6.004078041 | 1.92E-09 | 5.72E-08 |
| *ENSMUSG00000068614* | LPS | CON | 370.791961 | 926.7020318 | 599.6961078 | -1.13803497 | 0.29979589 | -3.796032597 | 0.00014703 | 0.001285091 |
| *ENSMUSG00000073879* | LPS | CON | 2.060536999 | 8.680078764 | 4.786230667 | -1.145290248 | 0.42988437 | -2.664182111 | 0.007717577 | 0.033937293 |
| *ENSMUSG00000095547* | LPS | CON | 5.140092551 | 18.33268096 | 10.57233483 | -1.147591805 | 0.419172494 | -2.737755507 | 0.006186005 | 0.028528755 |
| *ENSMUSG00000031217* | LPS | CON | 951.2609209 | 2128.427184 | 1435.976441 | -1.147638988 | 0.091106575 | -12.59666478 | 2.20E-36 | 2.19E-33 |
| *ENSMUSG00000020108* | LPS | CON | 202.6582657 | 476.5359559 | 315.4314322 | -1.150298742 | 0.21479562 | -5.355317486 | 8.54E-08 | 1.77E-06 |
| *ENSMUSG00000048572* | LPS | CON | 302.6826857 | 688.6935033 | 461.6283165 | -1.150323309 | 0.146436493 | -7.855441542 | 3.98E-15 | 2.96E-13 |
| *ENSMUSG00000106870* | LPS | CON | 2.784645824 | 8.432383707 | 5.110184952 | -1.151743753 | 0.370283657 | -3.110436366 | 0.001868111 | 0.010973945 |
| *ENSMUSG00000023046* | LPS | CON | 765.1490906 | 1756.919155 | 1173.525 | -1.152719928 | 0.167798229 | -6.869678757 | 6.43E-12 | 3.03E-10 |
| *ENSMUSG00000037989* | LPS | CON | 3.229482239 | 10.71923106 | 6.313496458 | -1.154274587 | 0.409922043 | -2.815839269 | 0.004865 | 0.023573111 |
| *ENSMUSG00000031965* | LPS | CON | 1.804919622 | 13.59437617 | 6.65940173 | -1.157466076 | 0.424666435 | -2.725588792 | 0.006418691 | 0.029366476 |
| *ENSMUSG00000022096* | LPS | CON | 71.10386824 | 167.2385803 | 110.6887497 | -1.157703887 | 0.19741053 | -5.864448498 | 4.51E-09 | 1.23E-07 |
| *ENSMUSG00000050445* | LPS | CON | 10.85885276 | 27.22339806 | 17.59719494 | -1.161184628 | 0.26770838 | -4.337498241 | 1.44E-05 | 0.000168731 |
| *ENSMUSG00000037887* | LPS | CON | 154.5985147 | 358.0975217 | 238.3922235 | -1.161898672 | 0.167993366 | -6.916336642 | 4.63E-12 | 2.24E-10 |
| *ENSMUSG00000004328* | LPS | CON | 19.08828001 | 49.02076069 | 31.41341911 | -1.165732568 | 0.305101599 | -3.82080124 | 0.000133019 | 0.001180917 |
| *ENSMUSG00000040488* | LPS | CON | 2163.043887 | 5058.451786 | 3355.270669 | -1.168421697 | 0.182092232 | -6.416647674 | 1.39E-10 | 5.08E-09 |
| *ENSMUSG00000039004* | LPS | CON | 1131.330334 | 2570.497103 | 1723.928415 | -1.170338505 | 0.090479282 | -12.93487838 | 2.86E-38 | 3.46E-35 |
| *ENSMUSG00000031618* | LPS | CON | 75.18572834 | 174.6994595 | 116.1619706 | -1.171983054 | 0.150900302 | -7.766605074 | 8.06E-15 | 5.91E-13 |
| *ENSMUSG00000091971* | LPS | CON | 112.5168593 | 274.980617 | 179.4137007 | -1.172629691 | 0.25119789 | -4.668151041 | 3.04E-06 | 4.31E-05 |
| *ENSMUSG00000047414* | LPS | CON | 74.45177406 | 180.3026014 | 118.0374088 | -1.173693861 | 0.230447118 | -5.093115804 | 3.52E-07 | 6.38E-06 |
| *ENSMUSG00000056313* | LPS | CON | 465.6933852 | 1093.146862 | 724.0565817 | -1.183815812 | 0.168850607 | -7.011024918 | 2.37E-12 | 1.19E-10 |
| *ENSMUSG00000033768* | LPS | CON | 37.70210327 | 88.23554424 | 58.50999073 | -1.186824495 | 0.150441958 | -7.888919501 | 3.05E-15 | 2.30E-13 |
| *ENSMUSG00000050830* | LPS | CON | 2.48226344 | 8.387046261 | 4.913644602 | -1.192625625 | 0.405496052 | -2.941152248 | 0.003269938 | 0.017316248 |
| *ENSMUSG00000090877* | LPS | CON | 150.8124001 | 394.5850843 | 251.1893877 | -1.199687872 | 0.297871115 | -4.027540144 | 5.64E-05 | 0.000558031 |
| *ENSMUSG00000020836* | LPS | CON | 10.07696343 | 25.57807063 | 16.45977228 | -1.200699169 | 0.269226748 | -4.459806385 | 8.20E-06 | 0.000103336 |
| *ENSMUSG00000061462* | LPS | CON | 13.49168966 | 47.7337391 | 27.59135707 | -1.203422441 | 0.410394333 | -2.932356378 | 0.003364005 | 0.01767068 |
| *ENSMUSG00000024076* | LPS | CON | 5.071038804 | 15.80628565 | 9.491434565 | -1.203673797 | 0.379090989 | -3.17515803 | 0.001497549 | 0.009126533 |
| *ENSMUSG00000106383* | LPS | CON | 1.872863502 | 6.884602714 | 3.936520824 | -1.204353912 | 0.405226976 | -2.972047726 | 0.002958207 | 0.015965076 |
| *ENSMUSG00000061718* | LPS | CON | 36.52777796 | 89.53192584 | 58.35301532 | -1.206542399 | 0.226617395 | -5.32413851 | 1.01E-07 | 2.07E-06 |
| *ENSMUSG00000038725* | LPS | CON | 43.4240014 | 147.9250509 | 86.45384532 | -1.206702427 | 0.403743382 | -2.98878565 | 0.002800886 | 0.0152669 |
| *ENSMUSG00000115149* | LPS | CON | 4.225967277 | 11.40234534 | 7.180946478 | -1.208086051 | 0.337957044 | -3.574673384 | 0.000350665 | 0.002707143 |
| *ENSMUSG00000032338* | LPS | CON | 3.256985588 | 10.67447581 | 6.311246268 | -1.208813774 | 0.386535932 | -3.12729988 | 0.001764199 | 0.01046894 |
| *ENSMUSG00000055430* | LPS | CON | 6.811802548 | 19.8077689 | 12.16308281 | -1.210530835 | 0.364835173 | -3.31802119 | 0.000906576 | 0.006068363 |
| *ENSMUSG00000015354* | LPS | CON | 960.3811381 | 2298.710023 | 1511.457738 | -1.211423781 | 0.164218532 | -7.376900577 | 1.62E-13 | 9.94E-12 |
| *ENSMUSG00000031825* | LPS | CON | 835.843412 | 2002.279554 | 1316.140647 | -1.212182021 | 0.165133242 | -7.340629962 | 2.13E-13 | 1.28E-11 |
| *ENSMUSG00000048814* | LPS | CON | 2.034479504 | 7.827371422 | 4.419787941 | -1.216680125 | 0.414830943 | -2.932954125 | 0.003357535 | 0.017657429 |
| *ENSMUSG00000026255* | LPS | CON | 2.739270667 | 9.680670299 | 5.597494045 | -1.219327537 | 0.400372803 | -3.04548043 | 0.002323089 | 0.013101231 |
| *ENSMUSG00000031966* | LPS | CON | 9.529599382 | 25.27992451 | 16.01502738 | -1.219989058 | 0.297021099 | -4.107415477 | 4.00E-05 | 0.000414051 |
| *ENSMUSG00000019787* | LPS | CON | 2.68406803 | 15.71964621 | 8.051659044 | -1.224273885 | 0.431854117 | -2.834924659 | 0.004583649 | 0.022506144 |
| *ENSMUSG00000052188* | LPS | CON | 7.773071305 | 20.49731336 | 13.01246509 | -1.225732643 | 0.302580955 | -4.050924632 | 5.10E-05 | 0.000511062 |
| *ENSMUSG00000031980* | LPS | CON | 3.934372343 | 13.98597599 | 8.073267961 | -1.227624598 | 0.406460431 | -3.02028071 | 0.002525405 | 0.013999708 |
| *ENSMUSG00000116617* | LPS | CON | 21.81538643 | 54.06912203 | 35.09633638 | -1.228937312 | 0.20583318 | -5.970550086 | 2.36E-09 | 6.83E-08 |
| *ENSMUSG00000042985* | LPS | CON | 168.9142444 | 539.9865585 | 321.7087267 | -1.230394133 | 0.383630297 | -3.207239218 | 0.001340155 | 0.008301802 |
| *ENSMUSG00000041644* | LPS | CON | 18.23852381 | 49.87510787 | 31.26535254 | -1.242387085 | 0.29169206 | -4.259242037 | 2.05E-05 | 0.000229354 |
| *ENSMUSG00000031494* | LPS | CON | 67.14490194 | 176.3902204 | 112.1282684 | -1.24720065 | 0.26773264 | -4.658381022 | 3.19E-06 | 4.50E-05 |
| *ENSMUSG00000016458* | LPS | CON | 6.870507619 | 25.75962308 | 14.64837869 | -1.250863434 | 0.410660082 | -3.045982523 | 0.002319213 | 0.013088093 |
| *ENSMUSG00000085272* | LPS | CON | 10.54655281 | 31.09909344 | 19.00936366 | -1.254529793 | 0.336237672 | -3.731080411 | 0.00019066 | 0.001600337 |
| *ENSMUSG00000038418* | LPS | CON | 295.9408831 | 773.4814157 | 492.5752201 | -1.254864857 | 0.252830152 | -4.963272165 | 6.93E-07 | 1.18E-05 |
| *ENSMUSG00000038403* | LPS | CON | 2.230294773 | 10.81374493 | 5.764656603 | -1.262888116 | 0.429720235 | -2.938861176 | 0.003294206 | 0.01740665 |
| *ENSMUSG00000028967* | LPS | CON | 941.4045816 | 2409.122203 | 1545.758896 | -1.263927394 | 0.217130822 | -5.821040891 | 5.85E-09 | 1.56E-07 |
| *ENSMUSG00000086868* | LPS | CON | 11.24901329 | 31.48067772 | 19.57969865 | -1.268654615 | 0.290408059 | -4.368524137 | 1.25E-05 | 0.000149138 |
| *ENSMUSG00000040896* | LPS | CON | 6.634471226 | 19.08744531 | 11.76216644 | -1.274412301 | 0.314387681 | -4.053633076 | 5.04E-05 | 0.000505477 |
| *ENSMUSG00000036062* | LPS | CON | 35.17276856 | 93.41291608 | 59.15400577 | -1.275751278 | 0.247223805 | -5.160309206 | 2.47E-07 | 4.63E-06 |
| *ENSMUSG00000117819* | LPS | CON | 3.176958597 | 15.04596423 | 8.064196211 | -1.280978517 | 0.42864588 | -2.988430726 | 0.002804141 | 0.015279723 |
| *ENSMUSG00000085730* | LPS | CON | 2.438437731 | 8.629889465 | 4.987859033 | -1.282346669 | 0.386850589 | -3.314837063 | 0.000916966 | 0.006119128 |
| *ENSMUSG00000031431* | LPS | CON | 1953.125724 | 4857.458162 | 3149.027316 | -1.284868621 | 0.128505114 | -9.998579714 | 1.55E-23 | 3.64E-21 |
| *ENSMUSG00000028862* | LPS | CON | 318.3252329 | 836.6557324 | 531.7554386 | -1.285011659 | 0.231978463 | -5.539357587 | 3.04E-08 | 7.04E-07 |
| *ENSMUSG00000046182* | LPS | CON | 12.91652184 | 36.21407765 | 22.50963305 | -1.285421373 | 0.298479868 | -4.306559715 | 1.66E-05 | 0.000191227 |
| *ENSMUSG00000115970* | LPS | CON | 8.36285782 | 22.10399743 | 14.02097413 | -1.29552589 | 0.259248228 | -4.997241051 | 5.82E-07 | 1.01E-05 |
| *ENSMUSG00000020067* | LPS | CON | 2.272671058 | 9.708893202 | 5.334644882 | -1.297588029 | 0.419605462 | -3.092400236 | 0.00198545 | 0.011531273 |
| *ENSMUSG00000020524* | LPS | CON | 60.34081844 | 162.2592871 | 102.3072467 | -1.300279104 | 0.240514279 | -5.406244932 | 6.44E-08 | 1.38E-06 |
| *ENSMUSG00000089712* | LPS | CON | 22.39107146 | 61.80633913 | 38.62088756 | -1.306661119 | 0.277500566 | -4.708679114 | 2.49E-06 | 3.59E-05 |
| *ENSMUSG00000041202* | LPS | CON | 5.242131782 | 15.53634445 | 9.480925235 | -1.310168445 | 0.314691408 | -4.163343555 | 3.14E-05 | 0.000333518 |
| *ENSMUSG00000051726* | LPS | CON | 3.393573586 | 11.91203925 | 6.901177096 | -1.312787298 | 0.375752827 | -3.493752284 | 0.000476283 | 0.003508906 |
| *ENSMUSG00000036913* | LPS | CON | 2.537395308 | 9.314944127 | 5.328150704 | -1.31451301 | 0.385196072 | -3.412581554 | 0.000643507 | 0.004564126 |
| *ENSMUSG00000021798* | LPS | CON | 26.49842808 | 84.08026703 | 50.20859706 | -1.322850281 | 0.350097831 | -3.778516069 | 0.000157766 | 0.001357892 |
| *ENSMUSG00000028341* | LPS | CON | 70.91736174 | 189.1481583 | 119.6006309 | -1.323463182 | 0.212189439 | -6.237177437 | 4.46E-10 | 1.48E-08 |
| *ENSMUSG00000023067* | LPS | CON | 1123.876587 | 2901.076033 | 1855.664594 | -1.327156979 | 0.147776215 | -8.980856485 | 2.69E-19 | 3.89E-17 |
| *ENSMUSG00000070529* | LPS | CON | 2.046021328 | 8.091994122 | 4.535539537 | -1.33715244 | 0.396533123 | -3.372107808 | 0.000745952 | 0.005148379 |
| *ENSMUSG00000042807* | LPS | CON | 395.8369723 | 1051.357737 | 665.7572871 | -1.33726007 | 0.18839547 | -7.098154048 | 1.26E-12 | 6.71E-11 |
| *ENSMUSG00000022456* | LPS | CON | 15.10141245 | 44.33290356 | 27.13790879 | -1.339255582 | 0.290730203 | -4.606523744 | 4.09E-06 | 5.62E-05 |
| *ENSMUSG00000031283* | LPS | CON | 60.48471055 | 165.1881994 | 103.5979119 | -1.377190926 | 0.184648073 | -7.458463569 | 8.75E-14 | 5.57E-12 |
| *ENSMUSG00000060913* | LPS | CON | 0.345431704 | 5.467898698 | 2.454682819 | -1.383549724 | 0.422365461 | -3.275717005 | 0.001053941 | 0.006857889 |
| *ENSMUSG00000105746* | LPS | CON | 2.243772716 | 8.241582842 | 4.713459238 | -1.385579352 | 0.372577573 | -3.718901656 | 0.000200091 | 0.001663821 |
| *ENSMUSG00000030399* | LPS | CON | 13.39142756 | 64.37390642 | 34.38421297 | -1.388014702 | 0.421927932 | -3.289696173 | 0.001002956 | 0.006590211 |
| *ENSMUSG00000020884* | LPS | CON | 8.191889781 | 33.82681326 | 18.74744651 | -1.394120562 | 0.405475912 | -3.438232758 | 0.000585524 | 0.004203954 |
| *ENSMUSG00000031543* | LPS | CON | 11.39081655 | 40.57815861 | 23.40913387 | -1.395470277 | 0.367885181 | -3.793222314 | 0.000148705 | 0.001297719 |
| *ENSMUSG00000055546* | LPS | CON | 3.44497215 | 14.46515697 | 7.98269531 | -1.402489729 | 0.401216431 | -3.495593952 | 0.000473008 | 0.003487815 |
| *ENSMUSG00000033863* | LPS | CON | 1063.95211 | 2885.546628 | 1814.020441 | -1.405901586 | 0.12977388 | -10.8334712 | 2.39E-27 | 1.06E-24 |
| *ENSMUSG00000097654* | LPS | CON | 56.24985447 | 158.0356666 | 98.16165947 | -1.411406229 | 0.176880457 | -7.979435685 | 1.47E-15 | 1.17E-13 |
| *ENSMUSG00000057716* | LPS | CON | 2.916008027 | 10.6962274 | 6.119627768 | -1.411973007 | 0.355314012 | -3.973873701 | 7.07E-05 | 0.000680864 |
| *ENSMUSG00000115240* | LPS | CON | 4.293554964 | 13.78576461 | 8.202111876 | -1.414207615 | 0.314929201 | -4.490557271 | 7.10E-06 | 9.04E-05 |
| *ENSMUSG00000029368* | LPS | CON | 1.710156 | 7.821938691 | 4.226772402 | -1.419516958 | 0.407897306 | -3.480084175 | 0.000501256 | 0.003667359 |
| *ENSMUSG00000044349* | LPS | CON | 26.51562294 | 77.95609578 | 47.69699411 | -1.42199453 | 0.245057142 | -5.802705919 | 6.53E-09 | 1.73E-07 |
| *ENSMUSG00000031442* | LPS | CON | 439.6285083 | 1222.28402 | 761.8984248 | -1.423494002 | 0.157930667 | -9.01341096 | 2.00E-19 | 2.97E-17 |
| *ENSMUSG00000032511* | LPS | CON | 22.18707189 | 63.85367182 | 39.34390715 | -1.431391739 | 0.203741797 | -7.025518359 | 2.13E-12 | 1.08E-10 |
| *ENSMUSG00000092035* | LPS | CON | 16.31892696 | 50.36756079 | 30.33895265 | -1.431865294 | 0.26821121 | -5.338573637 | 9.37E-08 | 1.92E-06 |
| *ENSMUSG00000025488* | LPS | CON | 4.525912748 | 17.41981649 | 9.83516723 | -1.441732585 | 0.385166741 | -3.743138829 | 0.000181736 | 0.001539163 |
| *ENSMUSG00000021750* | LPS | CON | 186.07864 | 552.2592518 | 336.8588919 | -1.447601514 | 0.230527275 | -6.279523806 | 3.40E-10 | 1.17E-08 |
| *ENSMUSG00000031340* | LPS | CON | 8.116790303 | 26.67932476 | 15.76018684 | -1.449753033 | 0.320527865 | -4.523017157 | 6.10E-06 | 7.96E-05 |
| *ENSMUSG00000038572* | LPS | CON | 7.063416423 | 25.35371962 | 14.59471774 | -1.462111103 | 0.348585678 | -4.194409569 | 2.74E-05 | 0.000296908 |
| *ENSMUSG00000004360* | LPS | CON | 23.02226443 | 67.52411809 | 41.34655711 | -1.462742339 | 0.211272374 | -6.923490805 | 4.41E-12 | 2.14E-10 |
| *ENSMUSG00000085139* | LPS | CON | 1.507389199 | 11.06322603 | 5.442145541 | -1.4630444 | 0.431895297 | -3.387497875 | 0.000705333 | 0.004914108 |
| *ENSMUSG00000027840* | LPS | CON | 17.23968292 | 55.55930542 | 33.01835101 | -1.465673139 | 0.286617753 | -5.113685828 | 3.16E-07 | 5.81E-06 |
| *ENSMUSG00000097789* | LPS | CON | 6.57980751 | 21.69419618 | 12.80337932 | -1.475872792 | 0.301334153 | -4.897794621 | 9.69E-07 | 1.58E-05 |
| *ENSMUSG00000112618* | LPS | CON | 1.955193371 | 7.893006319 | 4.400175173 | -1.497750591 | 0.370489209 | -4.042629455 | 5.29E-05 | 0.000526995 |
| *ENSMUSG00000057606* | LPS | CON | 89.52986249 | 307.9651461 | 179.4738028 | -1.530834854 | 0.30164629 | -5.074933475 | 3.88E-07 | 6.97E-06 |
| *ENSMUSG00000030087* | LPS | CON | 218.4345936 | 662.4350764 | 401.2583218 | -1.543449563 | 0.164710245 | -9.370695585 | 7.21E-21 | 1.23E-18 |
| *ENSMUSG00000020123* | LPS | CON | 3.84254527 | 14.51202326 | 8.235859738 | -1.561109784 | 0.33214775 | -4.700046241 | 2.60E-06 | 3.73E-05 |
| *ENSMUSG00000030428* | LPS | CON | 6.066846695 | 24.25291753 | 13.5552288 | -1.582124784 | 0.35189809 | -4.495974337 | 6.93E-06 | 8.85E-05 |
| *ENSMUSG00000003545* | LPS | CON | 22.39812866 | 83.0932398 | 47.39023324 | -1.590564789 | 0.314224728 | -5.061870201 | 4.15E-07 | 7.42E-06 |
| *ENSMUSG00000050505* | LPS | CON | 4.985193358 | 20.42401868 | 11.34235673 | -1.591181329 | 0.348657574 | -4.563736594 | 5.03E-06 | 6.70E-05 |
| *ENSMUSG00000052229* | LPS | CON | 1.403344467 | 9.759519419 | 4.844122389 | -1.601576571 | 0.423793686 | -3.779142122 | 0.00015737 | 0.001355755 |
| *ENSMUSG00000010651* | LPS | CON | 12.95301472 | 52.76828966 | 29.3475397 | -1.627340795 | 0.344054176 | -4.729896948 | 2.25E-06 | 3.29E-05 |
| *ENSMUSG00000028444* | LPS | CON | 13.43404223 | 49.42070295 | 28.252079 | -1.632918271 | 0.285185311 | -5.725814786 | 1.03E-08 | 2.63E-07 |
| *ENSMUSG00000022464* | LPS | CON | 83.55944081 | 285.0053423 | 166.5077532 | -1.64060653 | 0.222611224 | -7.369828437 | 1.71E-13 | 1.04E-11 |
| *ENSMUSG00000066687* | LPS | CON | 246.0815395 | 1407.315698 | 724.2367812 | -1.649313752 | 0.412102003 | -4.002197854 | 6.28E-05 | 0.000613791 |
| *ENSMUSG00000057933* | LPS | CON | 12.54615478 | 54.88990199 | 29.9818154 | -1.649611361 | 0.36249356 | -4.550732878 | 5.35E-06 | 7.07E-05 |
| *ENSMUSG00000020427* | LPS | CON | 519.7217615 | 1784.867391 | 1040.664079 | -1.660808806 | 0.217069495 | -7.651046521 | 1.99E-14 | 1.38E-12 |
| *ENSMUSG00000026077* | LPS | CON | 51.10337652 | 182.4603383 | 105.1915372 | -1.661471054 | 0.250429302 | -6.63449141 | 3.26E-11 | 1.34E-09 |
| *ENSMUSG00000004892* | LPS | CON | 7.001669123 | 29.61542248 | 16.31321462 | -1.677572185 | 0.33444001 | -5.01606307 | 5.27E-07 | 9.24E-06 |
| *ENSMUSG00000001494* | LPS | CON | 1.902722463 | 14.01251037 | 6.889105718 | -1.689217132 | 0.424425036 | -3.980012937 | 6.89E-05 | 0.000665529 |
| *ENSMUSG00000001670* | LPS | CON | 5.076990708 | 25.54814986 | 13.50629153 | -1.692612432 | 0.387801149 | -4.364640062 | 1.27E-05 | 0.000151386 |
| *ENSMUSG00000027442* | LPS | CON | 12.92665963 | 53.67524951 | 29.70549075 | -1.695010768 | 0.326165047 | -5.196788516 | 2.03E-07 | 3.90E-06 |
| *ENSMUSG00000032291* | LPS | CON | 1.481864345 | 8.800388487 | 4.495374286 | -1.725440113 | 0.401622708 | -4.296171705 | 1.74E-05 | 0.000198782 |
| *ENSMUSG00000022494* | LPS | CON | 2.494085281 | 12.33328341 | 6.545519806 | -1.754488821 | 0.366642157 | -4.785289381 | 1.71E-06 | 2.59E-05 |
| *ENSMUSG00000032315* | LPS | CON | 576.4144782 | 2395.671551 | 1325.520332 | -1.759472143 | 0.304889008 | -5.770861187 | 7.89E-09 | 2.06E-07 |
| *ENSMUSG00000050671* | LPS | CON | 2.091732685 | 10.59946074 | 5.594914825 | -1.77281083 | 0.36745257 | -4.824597709 | 1.40E-06 | 2.19E-05 |
| *ENSMUSG00000032773* | LPS | CON | 1.62980261 | 10.16469226 | 5.144168937 | -1.789802563 | 0.393198455 | -4.551906397 | 5.32E-06 | 7.04E-05 |
| *ENSMUSG00000013611* | LPS | CON | 1.085033788 | 8.876278399 | 4.293193334 | -1.810348529 | 0.419967902 | -4.310683082 | 1.63E-05 | 0.00018812 |
| *ENSMUSG00000028940* | LPS | CON | 7.217676744 | 30.26865086 | 16.70925432 | -1.81507841 | 0.293706547 | -6.179904487 | 6.41E-10 | 2.06E-08 |
| *ENSMUSG00000002588* | LPS | CON | 156.812771 | 661.9124922 | 364.7950091 | -1.857821787 | 0.266538731 | -6.970175715 | 3.17E-12 | 1.56E-10 |
| *ENSMUSG00000057074* | LPS | CON | 27.20508838 | 143.9749698 | 75.28680424 | -1.912830651 | 0.348488652 | -5.488932394 | 4.04E-08 | 9.16E-07 |
| *ENSMUSG00000054422* | LPS | CON | 12.1394786 | 59.25968535 | 31.54191668 | -1.935325063 | 0.305437892 | -6.336231072 | 2.35E-10 | 8.29E-09 |
| *ENSMUSG00000032419* | LPS | CON | 1.281340556 | 13.84920505 | 6.456343583 | -1.941287704 | 0.426241767 | -4.554428621 | 5.25E-06 | 6.97E-05 |
| *ENSMUSG00000022206* | LPS | CON | 1134.919965 | 5220.542398 | 2817.235084 | -2.060450343 | 0.212167675 | -9.711424415 | 2.70E-22 | 5.07E-20 |
| *ENSMUSG00000019577* | LPS | CON | 92.5535414 | 501.1534722 | 260.8005717 | -2.078324446 | 0.307008146 | -6.769606832 | 1.29E-11 | 5.74E-10 |
| *ENSMUSG00000031725* | LPS | CON | 16.67499684 | 108.7295066 | 54.57979497 | -2.157911838 | 0.346993243 | -6.2188872 | 5.01E-10 | 1.65E-08 |
| *ENSMUSG00000065987* | LPS | CON | 0.337388381 | 8.332256427 | 3.62939287 | -2.501941034 | 0.417506289 | -5.992582868 | 2.07E-09 | 6.08E-08 |
| *ENSMUSG00000034783* | LPS | CON | 0.959454793 | 19.70560314 | 8.678457055 | -3.183145513 | 0.362823453 | -8.773262817 | 1.74E-18 | 2.26E-16 |
