# Supplementary material for: Identification of robust genetic signatures associated with lipopolysaccharide-induced acute lung injury onset and astaxanthin therapeutic effects by integrative analysis of RNA sequencing data and GEO datasets
Source: Aging (Albany NY). 2020 Sep 23;12(18):18716–40. doi: 10.18632/aging.104042 (PMC7585091; doi:10.18632/aging.104042)
Supplement: Supplementary Tables 4, 5 and 6 [file aging-12-104042-s004..pdf]

## SUPPLEMENTARY TABLES

### Supplementary Table 4. DEGs between AST and LPS samples.

| Gene | sampleA | sampleB | baseMeanA | baseMeanB | baseMean | log2FoldChange | lfcSE | stat | pvalue | padj |
|---|---|---|---|---|---|---|---|---|---|---|
| ENSMUSG00000022818 | AST | LPS | 48.41487417 | 15.22940743 | 28.89401138 | 1.021436182 | 0.212138014 | 4.814960607 | 1.47229E-06 | 0.000673911 |
| ENSMUSG00000059824 | AST | LPS | 569.0902514 | 204.3558614 | 354.5406102 | 1.022655966 | 0.202188092 | 5.057943611 | 4.23802E-07 | 0.000231532 |
| ENSMUSG00000060600 | AST | LPS | 309.7794502 | 108.9422031 | 191.6398931 | 1.081034961 | 0.19750523 | 5.473449788 | 4.41358E-08 | 3.73742E-05 |
| ENSMUSG00000048498 | AST | LPS | 342.5834924 | 70.69719237 | 182.6503747 | 1.167546697 | 0.218068915 | 5.354026256 | 8.60184E-08 | 6.62185E-05 |
| ENSMUSG00000039109 | AST | LPS | 596.1148924 | 204.052468 | 365.4899369 | 1.167651944 | 0.188164286 | 6.205491853 | 5.45261E-10 | 9.23454E-07 |
| ENSMUSG00000032915 | AST | LPS | 613.660102 | 149.1405857 | 340.4133277 | 1.183303614 | 0.21566635 | 5.486732691 | 4.09436E-08 | 3.73742E-05 |
| ENSMUSG00000027338 | AST | LPS | 62.4055815 | 9.174534721 | 31.09320104 | 1.194972807 | 0.216388509 | 5.522348729 | 3.34498E-08 | 3.33238E-05 |
| ENSMUSG00000033227 | AST | LPS | 19.33965891 | 1.81051473 | 9.028397628 | 1.433440935 | 0.217019981 | 6.605110399 | 3.97221E-11 | 1.34547E-07 |
| ENSMUSG00000051682 | AST | LPS | 522.9979615 | 102.0594688 | 275.3870835 | 1.483269424 | 0.211237851 | 7.021797556 | 2.19032E-12 | 9.96623E-09 |

### Supplementary Table 5. GO analysis of the 11 AST-associated genes.

| ID | Description | BgRatio | pvalue | p.adjust | qvalue | geneID | Count |
|---|---|---|---|---|---|---|---|
| GO:0034364 | high-density lipoprotein particle | 23/23436 | 5.04E-05 | 0.000750507 | 0.000164585 | 20210/20208 | 2 |
| GO:0034358 | plasma lipoprotein particle | 34/23436 | 0.000111442 | 0.000750507 | 0.000164585 | 20210/20208 | 2 |
| GO:1990777 | lipoprotein particle | 34/23436 | 0.000111442 | 0.000750507 | 0.000164585 | 20210/20208 | 2 |
| GO:0032994 | protein-lipid complex | 36/23436 | 0.000125085 | 0.000750507 | 0.000164585 | 20210/20208 | 2 |
| GO:0020005 | symbiont-containing vacuole membrane | 12/23436 | 0.005619159 | 0.019216454 | 0.004214135 | 634650 | 1 |
| GO:0020003 | symbiont-containing vacuole | 14/23436 | 0.006552889 | 0.019216454 | 0.004214135 | 634650 | 1 |
| GO:0065010 | extracellular membrane-bounded organelle | 16/23436 | 0.007485823 | 0.019216454 | 0.004214135 | 634650 | 1 |
| GO:0030430 | host cell cytoplasm | 17/23436 | 0.007951991 | 0.019216454 | 0.004214135 | 634650 | 1 |
| GO:0033655 | host cell cytoplasm part | 17/23436 | 0.007951991 | 0.019216454 | 0.004214135 | 634650 | 1 |
| GO:0033646 | host intracellular part | 20/23436 | 0.009349301 | 0.019216454 | 0.004214135 | 634650 | 1 |
| GO:0043656 | intracellular region of host | 20/23436 | 0.009349301 | 0.019216454 | 0.004214135 | 634650 | 1 |
| GO:0033643 | host cell part | 22/23436 | 0.010279847 | 0.019216454 | 0.004214135 | 634650 | 1 |
| GO:0018995 | host | 24/23436 | 0.011209598 | 0.019216454 | 0.004214135 | 634650 | 1 |
| GO:0043657 | host cell | 24/23436 | 0.011209598 | 0.019216454 | 0.004214135 | 634650 | 1 |
| GO:0005881 | cytoplasmic microtubule | 78/23436 | 0.036014707 | 0.04801961 | 0.010530616 | 20208 | 1 |

**Supplementary Table 6. The list of primer sequences used in this study.**

| | | | | | |
|---|---|---|---|---|---|
| 1 | GAPDH | forward | 5′ - | AGGTCGGTGTGAACGGATTTG | - 3′ |
| | | reverse | 5′ - | TGTAGACCATGTAGTTGAGGTCA | - 3′ |
| 2 | Saa3 | forward | 5′ - | AGAGAGGCTGTTCAGAAGTTCA | - 3′ |
| | | reverse | 5′ - | AGCAGGTCGGAAGTGGTTG | - 3′ |
| 3 | Ly6i | forward | 5′ - | CTGAGAGGAACCCTTCTCCAA | - 3′ |
| | | reverse | 5′ - | GGTGTTCCACACACTACCCC | - 3′ |
| 4 | Saa1 | forward | 5′ - | TGGCTGGAAAGATGGAGACAA | - 3′ |
| | | reverse | 5′ - | AAAGCTCTCTCTTGCATCACTG | - 3′ |
| 5 | Irf7 | forward | 5′ - | GAGACTGGCTATTGGGGGAG | - 3′ |
| | | reverse | 5′ - | GACCGAAATGCTTCCAGGG | - 3′ |
| 6 | Cxcl5 | forward | 5′ - | GTTCCATCTCGCCATTCATGC | - 3′ |
| | | reverse | 5′ - | GCGGCTATGACTGAGGAAGG | - 3′ |
| 7 | Ccl7 | forward | 5′ - | GCTGCTTTCAGCATCCAAGTG | - 3′ |
| | | reverse | 5′ - | CCAGGGACACCGACTACTG | - 3′ |
| 8 | Timp1 | forward | 5′ - | CGAGACCACCTTATACCAGCG | - 3′ |
| | | reverse | 5′ - | ATGACTGGGGTGTAGGCGTA | - 3′ |
| 9 | Isg15 | forward | 5′ - | AGTGATGCTAGTGGTACAGAACT | - 3′ |
| | | reverse | 5′ - | CAGTCTGCGTCAGAAAGACCT | - 3′ |
| 10 | Gbp11 | forward | 5′ - | CATTGCAGAGAAGTGCACCAA | - 3′ |
| | | reverse | 5′ - | ATCACAGTCTCTCATTTGCTCC | - 3′ |
| 11 | Tgtp1 | forward | 5′ - | TGGGACCACTAACTTCACACC | - 3′ |
| | | reverse | 5′ - | GGCCAGTTGTGCATCATTTTC | - 3′ |
| 12 | Cxcl13 | forward | 5′ - | GGCCACGGTATTCTGGAAGC | - 3′ |
| | | reverse | 5′ - | GGGCGTAACTTGAATCCGATCTA | - 3′ |
